# Using resonance synchronous spectroscopy to characterize the reactivity and electrophilicity of biologically relevant sulfane sulfur

**DOI:** 10.1016/j.redox.2019.101179

**Published:** 2019-03-26

**Authors:** Huanjie Li, Huaiwei Liu, Zhigang Chen, Rui Zhao, Qingda Wang, Mingxue Ran, Yongzhen Xia, Xin Hu, Jihua Liu, Ming Xian, Luying Xun

**Affiliations:** a State Key Laboratory of Microbial Technology, Shandong University, 72 Binhai Road, Qingdao, 266237, People's Republic of China; b Institute of Marine Science and Technology, Shandong University, 72 Binhai Road, Qingdao, 266237, People's Republic of China; c Department of Chemistry, Washington State University, Pullman, WA, 99164-4630, USA; d School of Molecular Biosciences, Washington State University, Pullman, WA, 99164-7520, USA

## Abstract

Sulfane sulfur is common inside cells, playing both regulatory and antioxidant roles. However, there are unresolved issues about its chemistry and biochemistry. We report the discovery that reactive sulfane sulfur such as polysulfides and persulfides could be detected by using resonance synchronous spectroscopy (RS_2_). With RS_2_, we showed that inorganic polysulfides at low concentrations were unstable with a half-life about 1 min under physiological conditions due to reacting with glutathione. The protonated form of glutathione persulfide (GSSH) was electrophilic and had RS_2_ signal. GSS^−^ was nucleophilic, prone to oxidation, but had no RS_2_ signal. Using this phenomenon, p*K*_a_ of GSSH was determined as 6.9. GSSH/GSS^−^ was 50-fold more reactive than H_2_S/HS^−^ towards H_2_O_2_ at pH 7.4, supporting reactive sulfane sulfur species like GSSH/GSS^−^ may act as antioxidants inside cells. Further, protein persulfides were shown to be in two forms: at pH 7.4 the deprotonated form (R-SS^-^) without RS_2_ signal was not reactive toward sulfite, and the protonated form (R-SSH) in the active site of a rhodanese had RS_2_ signal and readily reacted with sulfite to produce thiosulfate. These data suggest that RS_2_ of sulfane sulfur is likely associated with its electrophilicity. Sulfane sulfur showed species-specific RS_2_ spectra and intensities at physiological pH, which may reveal the relative abundance of a reactive sulfane sulfur species inside cells.

## 1 Introduction

Hydrogen sulfide (H_2_S) is a new gasotransmitter that serves many important regulatory roles in biological systems [1]. H_2_S is involved in vascular homeostasis, neurological function, cytoprotection, anti-inflammation, and revascularization [[1], [2], [3]]. However, accumulating evidences imply that H_2_S is converted to reactive sulfane sulfur, which plays the observed roles [[4], [5], [6]]. Reactive sulfane sulfur includes organic persulfides (R-SSH), organic polysulfides (R-SS_n_H or R-SS_n_R, n ≥ 2), and inorganic hydrogen polysulfides (H_2_S_n_, n ≥ 2) [7]. Reactive sulfane sulfur is different from thiols, as it often possesses both nucleophilic and electrophilic characteristics while thiols mainly function as nucleophiles [8]. The reactive sulfane sulfur can be produced from specific and nonspecific enzymatic oxidations of H_2_S [9,10] or from the metabolism of cysteine and *N*-Acetyl cysteine (NAC) [[11], [12], [13]]. GSSH is a key form of reactive sulfane sulfur in the sulfide oxidation pathway of heterotrophic bacteria and human mitochondria [14,15]. Reactive sulfane sulfur can modify cysteine residues in a large number of proteins by S-persulfidation (R-SSH), which can alter enzyme activity and influence biological processes via signaling [13,16]. For instance, rhodanese (thiosulfate:cyanide sulfurtransferase) that is present in almost all living organisms catalyzes the transfer of the sulfane sulfur from thiosulfate to cyanide via an intermediate (R-SSH) at its catalytic Cys residue [17,18]. Collectively, previous reports have revealed the significance of reactive sulfane sulfur in biological processes. Thus, a better understanding of the chemical and biochemical properties of biologically relevant reactive sulfane sulfur will help to advance the field [19,20].

Current methods used for the detection of reactive sulfane sulfur include sulfur chemiluminescence detection, ion chromatography, HPLC analysis of the monobromobimane derivative of H_2_S_n_, and the use of H_2_S_n_-sensitive fluorescent dyes in living cells or *in vitro* [5,7,21]. Gao et al. developed some fluorescent probes that serve as an effective imaging tool for tracing or monitoring concentration changes of endogenous sulfane sulfur [22,23]. All of these methods are reaction-based. A reaction-free method that can real-timely probe reactive sulfane sulfur has not been developed. Here, we report the discovery that reactive sulfane sulfur can be detected via resonance synchronous spectroscopy (RS_2_) with a conventional spectrofluorometer by simultaneously scanning the excitation and emission (i.e. Δ*λ* = *λ*_em_–*λ*_ex_) [24]. This method is simple, fast, and nonintrusive for reactive sulfane sulfur analysis, allowing us to distinguish the protonated and deprotonated forms of persulfides and their reactivity.

## 2 Materials and methods

### 2.1 Materials and reactive sulfane sulfur preparations

Sodium hydrosulfide (NaHS), reduced glutathione (GSH), oxidized glutathione (GSSG), cysteine, cystine, thiosulfate, tetrathionate, bis[3-(triethoxysilyl)propyl] tetrasulfide (Tsp-SSSS-Tsp), bis(prop-2-en-1-yl) tetrasulfide (Pey-SSSS-Pey) were purchased from Sigma-Aldrich; dimethyl trisulfide (Me-SSS-Me) was purchased from TCI Company (Shanghai). Preparation of H_2_S_n_, existing as HS_n_H, HS_n_^−^, and S_n_^2−^ depending on the pH, was performed as a previous report [10]. GSSSG was prepared by following the protocol of Moutiez et al. [25]. Glutathione persulfide (GSSH) was obtained via reacting GSSG with sulfide, following the protocol of Luebke et al. [26]. The obtained products were confirmed by HPLC-fluorescence and MS analysis.

### 2.2 RS_2_ analysis of reactive sulfane sulfur

RF-5301 PC Spectrofluoro Photometer (SHIMADZU) was used to measure the fluorescence. Sample was diluted into 2 ml argon-deoxygenated buffers (Tris-HCl 50 mM, pH 7.4) in a parafilm-sealed fluorometer cell (d = 1 cm). Cluster 5 chemicals were dissolved in acetone to make a 100 mM stock and then diluted into argon-deoxygenated buffer. RS_2_ was acquired by simultaneously scanning the excitation (*λ*_ex_) and emission (*λ*_em_) on monochromators setting the offset (Δλ = *λ*_em_–*λ*_ex_) to a constant [27]. All spectra were acquired with a scan rate of 60 nm/min. The measurement interval was 1.0 nm and slit width was 5 nm. For pH relevant RS_2_ analysis, the concentrations of reactive sulfane sulfur were carefully selected to let the RS_2_ intensities fell into the detection range of RF-5301. Known amounts of H_2_S_n_ and GSSH were dissolved in 20 ml of 50 mM Tris-HCl solutions (pH 7.4) and 20 mM sodium phosphate solution (pH 6), respectively, and then were titrated with 500 mM NaOH via 10-μl additions. The solution mixture was vortexed, followed with pH measurement and RS_2_ acquisition. The RS_2_ intensities were used for determining p*K*_a_.

### 2.3 pK_a_ determination method

The average signal intensities of GSSH (375 nm–384 nm) and DUF442-C34-SSH (444 nm–453 nm) were used for determining their p*K*_a_ values, respectively. The p*K*_a_ calculating equation is deduced as below:

R_2_S_2_ (*I*_*R2S2*_^*obs*^) was obtained by dividing the observed *RS*_2_ intensity (*I*_*RS2*_^*solu*^) with the *RS*_*2*_ intensity of the buffer (*I*_*RS2*_^*solv*^).

(1) $$I_{{\text{R}}_{2}{\text{S}}_{2}}^{\text{obs}}\left(\text{λ}\right)=\frac{I_{{\text{RS}}_{2}}^{\text{solu}}}{I_{{\text{RS}}_{2}}^{\text{solv}}}$$

For RSSH/RSS^−^ mixture, the R_2_S_2_ is equal to that of fully protonated form (*RS*_*p*_) times the fraction of its protonated form (*f*_*p*_).

(2) $$R_{2}S_{2}=RS_{p}\times f_{p}$$

This equation can be rewritten as follow:

(3) $$R_{2}S_{2}=RS_{p}\times RSSH/\left(RSSH+RSS^{-}\right)$$

According to the Henderson-Hasselbalch equation, *p* is hill slop; the fill status is succeeded (100).

(4) $$RSSH/RSS^{-}=10ˆ\left(pK_{a}-pH\right)\times p$$

Substituting the right-hand side of eq. (4) into eq. (3), we obtain:

(5) $$R_{2}S_{2}=RS_{p}/\left(1+10ˆ\left(pH-pK_{a}\right)\times p\right)$$

The detected R_2_S_2_ intensity data of RSSH at different pH values were fitted with eq. (5) to obtain the p*K*_a_ value.

### 2.4 ^1^H NMR and ^13^C–^1^H HMQC analysis

The ^1^H NMR spectra were recorded on a Bruker spectrometer at 600 MHz with a 5-mm probe. ^13^C–^1^H HMQC spectra were recorded on the Bruker spectrometer at 600 MHz with a 5-mm-gradient salt-tolerant H/C probe. The pulse sequence was set according to a previous report [28]. Delay = 1.5s, Size of fid = 1024, Number of scans = 64. The NMR data were processed and analyzed with Mestrelab Mnova version 10.

### 2.5 Chemical reactions analysis

For RS_2_ analysis of GSSH disproportionation, 50 μM of GSSH was transferred into 20 mM sodium phosphate buffer of different pH, and RS_2_ was measured at selected time points as mentioned in the text. The reaction mixtures were also analyzed by HPLC-fluorescence and MS analysis.

For kinetics analysis, reactions were conducted in a fluorometer cell (d = 1 cm) sealed with parafilm. Reactions of H_2_S_n_ with GSH were performed in deoxygenated HEPES buffer (100 mM, pH 7.4), started by adding 200 μM–5 mM of GSH to 10 μM of H_2_S_n_. RS_2_ of 535 nm–545 nm was scanned immediately at 30-s intervals for 3 min. Reactions of RS_n_R with GSH were performed in deoxygenated HEPES buffer (100 mM, pH 7.4), started by adding 10 mM–20 mM of GSH to 500 μM of RS_n_R. RS_2_ of 535 nm–545 nm was scanned at 1-min intervals for 8 min. The *k*_*obs*_ value was calculated by plotting the *ln*_*[RS2]*_ value against the reaction time. The apparent 2^nd^-order reaction rate constant *k* was calculated with the formula: *k*_*obs*_* = k × [GSH]*. For H_2_S release detection, these reactions were performed in sealed tubes. Lead acetate papers were fixed in the gas phase of the tubes containing the reaction mixture. Reactions of DTT with H_2_S_n_ and RS_n_R were similar to the GSH reaction above, and the calculations were also similar.

Reactions of antioxidants (H_2_S or GSSH) with H_2_O_2_ were conducted in deoxygenated HEPES buffer (100 mM, pH 7.4), started by adding 50 μM–500 μM of the antioxidant to 50 μM of H_2_O_2_. H_2_O_2_ reacted with GSSH to generate GSSSG [29], which had RS_2_. The RS_2_ (535–545 nm) intensity of GSSSG was obtained and used to calculate the reaction rate. H_2_O_2_ reacted with H_2_S to generate H_2_S_2_, which displayed RS_2_, and the RS_2_ increase was used to obtain the reaction constant. The *k*_*obs*_ value was calculated by plotting the *L*_*n*_[GSSSG] (or *L*_*n*_[H_2_S_2_]) value against the reaction time. The apparent 2^nd^-order reaction rate constant *k* was calculated using the formula: *k*_*obs*_* = k × [antioxidant]*.

### 2.6 Protein purification and modification with GSSH

The DUF442 domain of SQR (GenBank accession number: AAZ62946.1) was cloned from *Cupriavidus pinatubonensis* JMP134. Site-directed mutagenesis was performed according to a revised method [30]. For protein expression, these genes were ligated into the pET30a vector with a His tag at the C-terminus and then expressed in *Escherichia coli* BL21 (DE3) (Table S1). The recombinant *E. coli* was grown in LB at 30 °C with shaking until OD_600__nm_ reached about 0.6, and 0.3 mM IPTG was added; the cells were further cultivated at 20 °C for 20 h. Cells were harvested and disrupted with crusher SPCH-18 (STANSTED); protein purification was carried out with nickel-nitrilotriacetic acid agarose resin (Invitrogen). Buffer exchange of the purified proteins was performed via PD-10 desalting column (GE Healthcare). The finally obtained protein was in HEPES buffer (25 mM, pH 8.0) containing 300 mM NaCl.

The purified protein (6.0 mg/ml) was mixed with 200 μM of GSSH in HEPES buffer (100 mM, pH 7.4). After incubated at 25 °C for 20 min, the mixture was loaded onto a PD-10 desalting column to remove small molecules. The re-purified protein was then subjected to LC-MS/MS, RS_2_ or sulfite reaction analysis. For RS_2_ analysis, the protein was diluted to 0.1–0.5 mg/ml in the HEPES buffer so that the RS_2_ intensities were within the detection range of our fluorometer (RF-5301). For protein-SSH p*K*_a_ determination, we diluted DUF442-C34-SH (the C94S mutant) and GSSH reacted-DUF442-C34-SSH in HEPES buffers of different pH (3, 3.5, 4, 4.5 … …6.5, 7, 7.4), and then detected their RS_2_ intensities. The p*K*_a_ was determined using Eq. (5). Titrating HCl solution into a protein solution may cause protein denaturation.

### 2.7 HPLC-fluorescence and MS analysis of persulfides

LC-fluorescence and MS analysis of GSSH and protein-SSH was performed by following a previously reported protocol [10]. Briefly, samples were derivatized with monobromobimane (mBBr) and were injected onto a C18 reverse phase column (VP-ODS, 150 × 4 mm, Shimadzu). The column was maintained at 30 °C and eluted with a gradient of solution A (0.25% acetic acid) and solution B (0.25% acetic acid and 75% methanol) in distilled water from 5% B to 70% B in 8 min, 70% B for 8 min, 100% B for 8 min at a flow rate of 0.8 ml/min. The fluorescence detector (LC-20A) was used for detection with excitation at 370 nm and emission at 485 nm. The ESI mass spectrometer (Ultimate 3000, Burker impact HD) was used with the source temperature at 200 °C and the ion spray voltage at 4.5 kV. Nitrogen was used as the nebulizer and drying gas.

### 2.8 HPLC analysis of H_2_S_n_

H_2_S_n_ (5 mM) was diluted in Tris-HCl buffer at different pH, derivatized with methyl trifluoromethanesulfonate (methyl triflate) and analyzed by reversed-phase liquid chromatography using a C18 reverse phase column (VP-ODS, 150 × 4 mm, Shimadzu) and eluted with pure methanol. HPLC analysis and peak position of dimethylpolysulfides from Me_2_S_2_ to Me_2_S_8_ and S_8_ were found from calibration curves according to a published protocol [31].

### 2.9 Bioinformatics analysis and protein structure modeling

The three-dimensional structure of DUF442 was generated by SWISS-MODEL (http://swissmodel.expasy.org/) and analyzed by PyMOL-1.5.0.3. Rhodanese from *Neisseria meningitidis* z2491 (PDB ID: 2F46) at 1.41 Å resolution was chosen as the template (39% sequence similarity). The global QMEAN score was −0.56 for the DUF442 model. Their catalytic cysteine residues and their nearby residues also showed high reliability scores. The surface electrostatic potentials were analyzed by APBS-1.1.0, and the data and parameters were obtained with the PDB2PQR server (http://nbcr-222.ucsd.edu/pdb2pqr_2.1.1/).

### 2.10 Detection of sulfane sulfurs by using SSP4

Reactions of GSSH with SSP4 (Sulfane Sulfur Probe 4, Dojindo China Co., Ltd) were conducted by mixing 10 μM of SSP4 with 20 μM of GSSH in 100 μl of HEPES (0.1 M) buffer at different pH. The mixture was incubated at room temperature for 30 min, and then the fluorescence was detected by using Synergy H1 microplate reader. The excitation wavelength was set at 482 nm and the emission wavelength was set at 515 nm.

### 2.11 Whole cell analysis by RS_2_

Wild-type *E. coli* BL21 and recombinant *E. coli* strain containing pBBR1-CpSQR were used for intracellular polysulfides analysis. The strain was incubated at 37 °C until OD_600nm_ reached about 0.6 in LB medium. To induce CpSQR expression, 0.3 mM IPTG was added, and the cells were further cultivated at 30 °C for 5 h. Cells were collected by centrifugation and washed twice with Tris buffer (50 mM, pH 7.4). Different concentrations of NaHS were added to cell suspension of 0.1 OD_600nm_. H_2_S oxidation was performed at 30 °C for 40 min. Then cells were collected and washed with Tris-HCl buffer (50 mM, pH 7.4). For RS_2_ analysis, cell intensity was adjusted to 0.01 OD_600nm_ in Tris-HCl buffer. Wild-type *E. coli* BL21 was incubated in LB medium at 37 °C. Cells were collected, washed twice with Tris-HCl buffer (50 mM, pH 7.4), and resuspended to 0.01 OD_600nm_ before RS_2_ analysis.

### 2.12 Whole cell analysis by SSP4

Wild-type *E. coli* BL21 cells were collected, washed twice with PBS buffer, and resuspended with PBS at 0.1 OD_600nm_. SSP4 (10 μM) and CTAB (0.5 mM) were added to the cell suspension and incubated for 15 min at room temperature. After centrifugation (4000 rpm, 5 min), the supernatant was discarded and remaining cells were washed twice with PBS buffer. The cells were diluted to 0.1 OD_600nm_ in PBS buffer. The fluorescence was analyzed by using Synergy H1 microplate reader.

## 3 Results

### 3.1 Discovery of strong RS_2_ in H_2_S_n_ and RS_n_R compounds

**Fig. 1:**
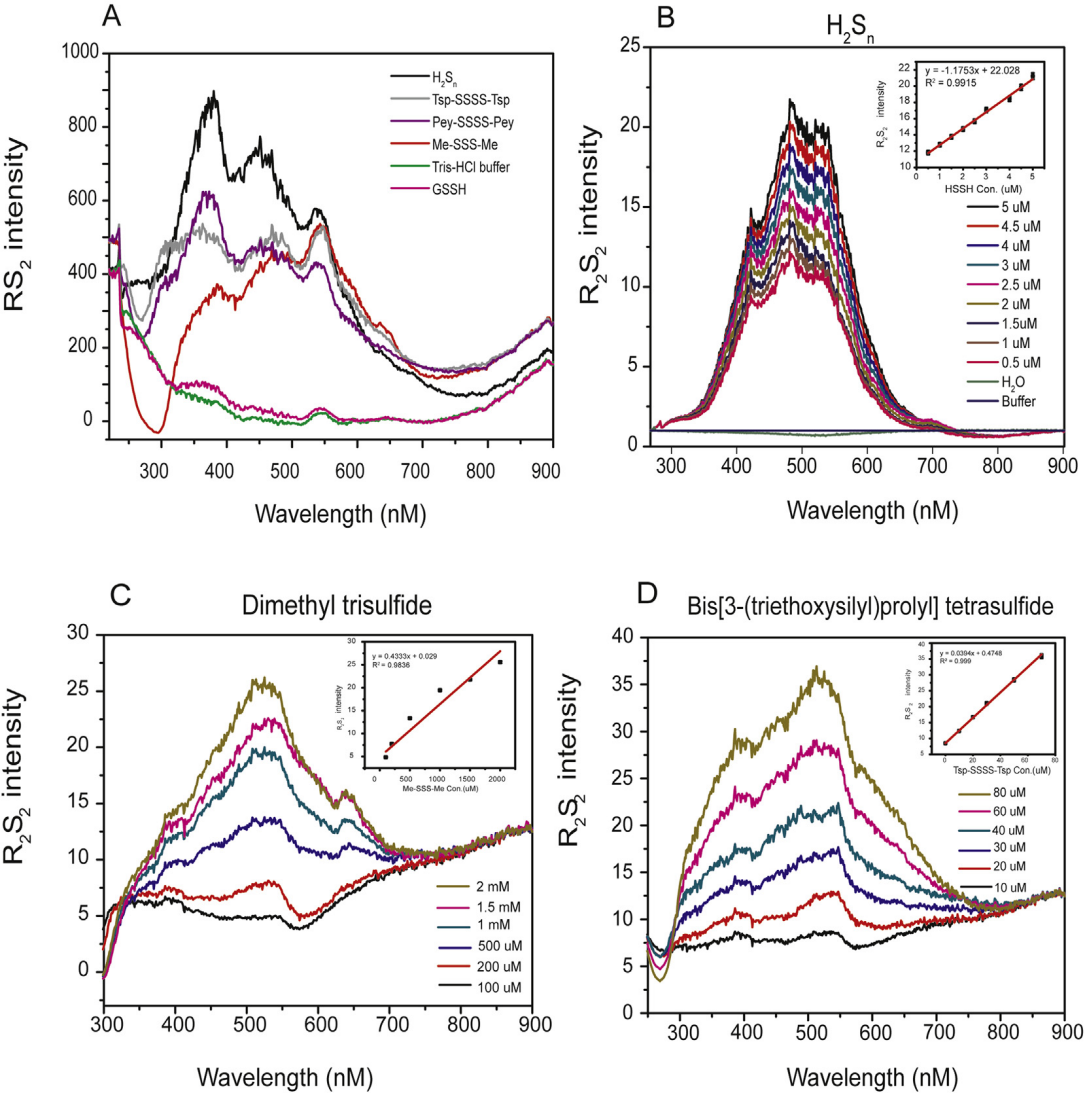
**RS**_**2**_**analysis of reactive sulfane sulfur and related compounds.** The compounds were diluted in Tris-HCl buffer (50 mM, pH 7.4) and analyzed with a fluorometer. (A) RS_2_ intensity curves of selected compounds from the RF-5301 PC Spectrofluoro Photometer. (B) H_2_S_n_ displayed a concentration-dependent, linear response to RS_2_ analysis when the solvent RS_2_ intensity was corrected; R_2_S_2_ values were obtained according to Eq. (1). (C–D) Me-SSS-Me and Tsp-SSSS-Tsp also showed good R_2_S_2_ responses to their concentrations.

When analyzing H_2_S_n_ using resonance synchronous spectroscopic (RS_2_) [21], we found that it had strong fluorescence intensity. Then we set the offset (Δ*λ* = *λ*_em_–*λ*_ex_) to a constant between the excitation and detection wavelength, i.e. Δ*λ* = 0,1,2 … 6 nm to scan the sample. The fluorescence intensity was the highest when Δ*λ* = 1 nm and decreased along with Δ*λ* increased. Thus, Δ*λ* = 1 nm was used for all analyses. Distilled water and 50 mM Tris-HCl had low RS_2_, and we used the Tris-HCl or HEPES buffer (pH 7.4) for most analyses (Fig. 1A). To test whether it is a common property of sulfur-containing compounds, we totally analyzed 14 sulfur-containing chemicals that were sorted into 7 clusters (Table 1). Among them, clusters 2 and 3 are important cellular persulfides and polysulfides; cluster 5 contains diallyl polysulfides (RS_n_R); other clusters are not polysulfides but are all involved in polysulfide metabolism. All chemicals were tested at pH 7.4. In addition to H_2_S_n_, Bis[3-(triethoxysilyl)propyl], tetrasulfide (Tsp-SSSS-Tsp) and dimethyl trisulfide (Me-SSS-Me) also showed significant RS_2_ (Fig. 1A).

**Table 1:** RS_2_ analysis of sulfur-containing chemicals.

Cluster	Containing group	Compound	RS_2_^d^
1	R-S-H	Cys-SH, GSH, NaHS	–
2	H_2_S_n_(n ≥ 2)	Polysulfides	++
3	R-SS-H	GSS^-^	–e
4	R-S_2_-R	GSSG, Cys-SS-Cys	–
5	R-S_n_-R	GSSSG	+
		Me-SSS-Me^a^	+
		Tsp-SSSS-Tsp^b^	+
		Pey-SSSS-Pey^c^	+
6	-S=O	SO_3_^2−^, SO_4_^2-^	–
7	-S-S=O	S_2_O_3_^2−^, S_4_O_6_^2-^	–

a Me is methyl group.

b Bis[3-(triethoxysilyl)propyl] tetrasulfide.

c Bis(prop-2-en-1-yl) tetrasulfide.

d – denotes no RS_2_ was detected even at 10 mM, ++ denotes RS_2_ was detected at 1 μM–20 μM, + denotes RS_2_ was detected at 10 μM–500 μM. RS_2_ was measured in 50 mM Tris buffer, pH 7.4.

e GSSH had strong RS_2_ at pH 6.

We diluted different concentration of H_2_S_n_ and cluster 5 compounds to analyze the RS_2_ intensity. The RS_2_ detection range was 0.2 μM–20 μM for H_2_S_n_ at pH7.4 and 10 μM–2 mM for cluster 5 compounds (Me-SSS-Me, 100 μM–2 mM; Tsp-SSSS-Tsp, 10 μM–80 μM). To remove the interference of the buffer, the ratiometric resonance synchronous spectroscopy (R_2_S_2_) value was obtained via dividing the sample RS_2_ intensity (I_RS2_^solu^) with the solvent RS_2_ intensity (I_RS2_^solv^) (Eq. (1) in methods) (Fig. 1) [27].

The R_2_S_2_ intensity of H_2_S_n_ showed good responses to its concentrations and followed linear dependence at fixed pH values (Fig. 1B, Figs. S1A and S1B). Me-SSS-Me and Tsp-SSSS-Tsp also showed good responses to its concentrations and followed linear dependence (Fig. 1C and D). For R_2_S_2_ detection of GSSSG, we only showed the range 0.025 μM–0.5 μM, but the response is linear up to 5 μM or higher (Fig. S1C). These results indicated RS_2_ is not a common property of all sulfur-containing chemicals, but a particular property of some chemicals that contain multiple sulfur atoms (n ≥ 2).

### 3.2 The pH effect on RS_2_ of reactive sulfane sulfur and its applications

**Fig. 2:**
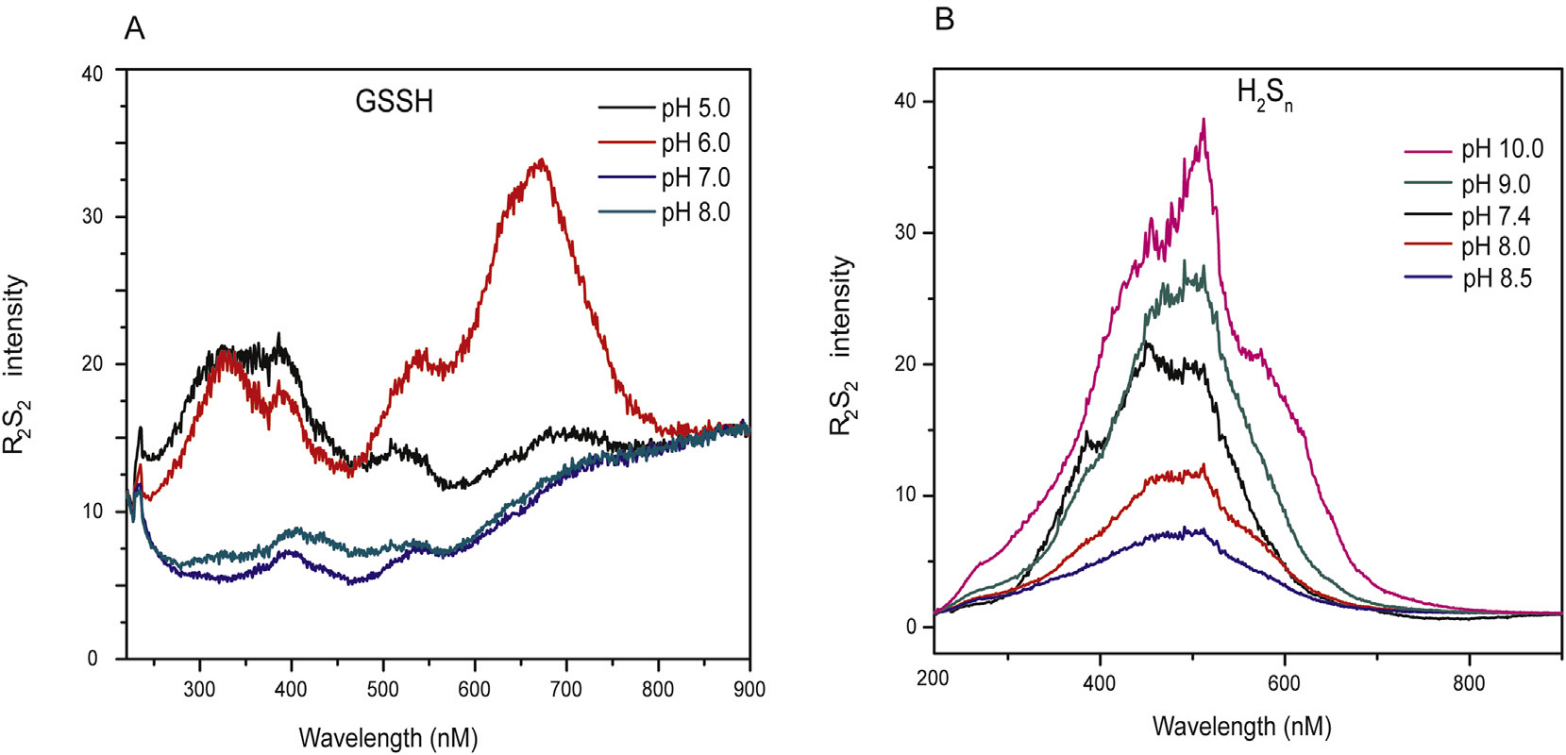
**The** pH **effect on RS**_**2**_**analysis of GSSH and H**_**2**_**S**_**n**_. (A) GSSH showed strong R_2_S_2_ at low pH. (B) The R_2_S_2_ intensity of H_2_S_n_ could be affected by different pH range. GSSH (20 μM) and H_2_S_n_ (5 μM) were diluted in 20 mM phosphate buffer (pH 5.0, 6.0, 7.0, 8.0) or 50 mM Tris buffer (7.4, 8.0, 8.5, 9.0, 10.0). The buffers had the same low RS_2_ intensity as that of water (data not shown). The RF-5301 PC spectrofluoro photometer was used for all analyses.

Since GSSH is a pivotal intermediate in cellular reactive sulfane sulfur metabolism [29], it was a surprise that RS_2_ of GSSH was hardly detectable at pH 7.4 (Fig. 1A). When we analyzed GSSH at different pH, it showed clear R_2_S_2_ at lower pH, especially at pH ≤ 6.0 (Fig. 2A). The highest peak was around 300 nm, which is consistent with its absorbance peak at 300 nm [10].

**Fig. 3:**
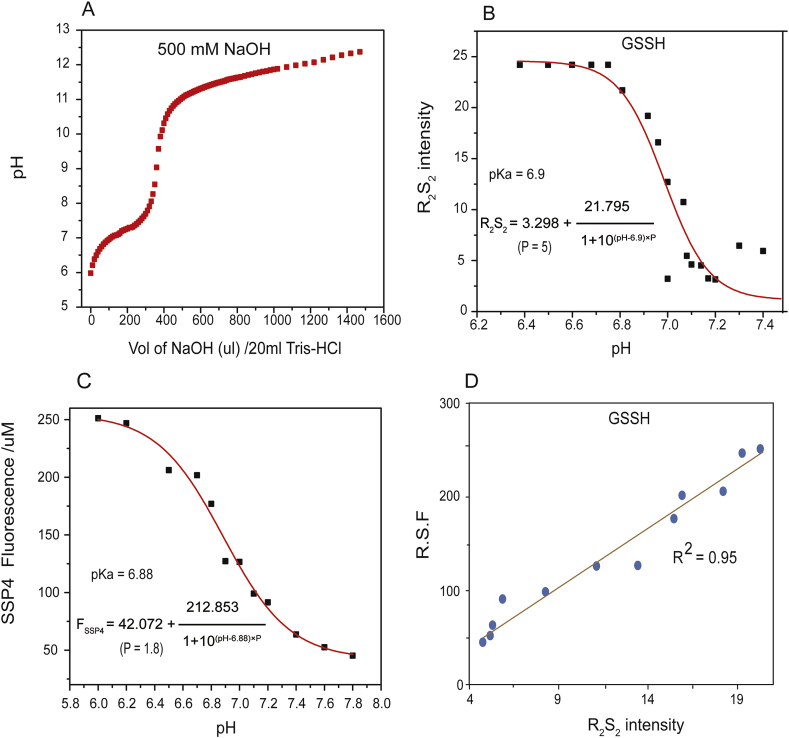
**p*K***_**a**_**determination of GSSH by using RS**_**2**_**and reaction with SSP4.** Deduction of the equation is shown in Method 2.3. (A) Solution pH as a function of titrant volume (μl) for 20 ml of 20 μM GSSH. (B) The R_2_S_2_ values of the GSSH solution at different pH. The data were used to calculate p*K*_a_ of GSSH. (C) The fluorescence of SSP4 after reacting with GSSH at different pH. The data also used to calculate p*K*_a_ of GSSH. (D) Electrophilicity shown as relative SSP4-induced fluorescence (R.S.F) after 30 min of reaction and R_2_S_2_ values of GSSH are well correlated.

We then used R_2_S_2_ to determine p*K*_a_ of GSSH/GSS^−^. GSSH was dissolved in 20 ml aliquots of 50 mM Tris-HCl solution (pH 6). The solution was titrated with NaOH, followed with pH measurement and RS_2_ acquisition (375 nm–384 nm). The p*K*_a_ value was determined as 6.9 via data fitting by using the Henderson-Hasselbach derived equation (Fig. 3B). GSSH can react with non-fluorescent SSP4 to release fluorescent fluorescein [7]. When GSSH and SSP4 were mixed at different pH, the reaction was rapid at pH 6 but not at pH 9.5 (Fig. S2). The results were logical from a chemical perspective, as GSSH is the electrophile and SSP4 is the nucleophile in the reaction. GSS^−^ should be more nucleophilic, while GSSH should be more electrophilic. Therefore, the reaction of SSP4 with GSSH should be faster than that with GSS^−^. The reaction rates at different pH were determined. The data were fitted with the Henderson-Hasselbach derived equation to obtain the estimated p*K*_a_ of GSSH. The value was 6.9, the same as that determined via R_2_S_2_ (Fig. 3C). Interestingly, the SSP4 reaction rates and R_2_S_2_ intensities at different pH were highly correlated (Fig. 3D), indicating that RS_2_ correlates with the electrophilicity of GSSH.

The pH change did not show apparent effect to RS_2_ of class 5 chemicals (RS_n_R) (data not shown). This is expected as RS_n_R compounds have no conditional protonation issues. When H_2_S_n_ in Tris buffer at pH 7.4 was titrated with 500 mM NaOH, the RS_2_ intensity decreased and reached the lowest level at pH around 8.5. However, RS_2_ increased again when more NaOH was added into the H_2_S_n_ solution. To confirm those results, we diluted H_2_S_n_ in Tris buffers of different pH (7.4, 8.0, 8.5, 9.0, and 10). The R_2_S_2_ intensity was high at pH 7.4 and low at pH 8.5 (Fig. 2B); R_2_S_2_ increased again at pH 9.0 and pH 10, but the spectrum changed similarly to that of Tsp-S_4_-Tsp (Fig. 1D). When H_2_S_n_ was derivatized and analyzed by HPLC, the chain length distribution at various pH corresponded well to the calculated equilibrium distribution of polysulfide ions in aqueous solutions of different pH [31]. At pH 7.4, most H_2_S_n_ was detected as S_8_, and a small peak of S_2_^2−^ was also detectable (Fig. S3A). At low concentrations such as 10 μM, H_2_S_2_/HS_2_^−^ has been detected as the dominant species [10,32]. With pH increased to 8.0 and 8.5, S_8_ gradually decreased, and S_2_^2−^, S_3_^2−^, S_4_^2−^, S_5_^2−^, S_6_^2−^, S_7_^2−^, and S_8_^2−^ were all detectable with S_2_^2−^ being the main species (Fig. S3B). At pH 9.0 and 10.0, S_8_ became a minor species, and S_5_^2−^, S_6_^2−^, and S_7_^2−^ were the dominant species (Fig. S3A). Large portions of S_8_ were detected in most samples except at pH 10 (Fig. S3), and we believe that this is likely due to the high concentration (5 mM) of H_2_S_n_ used in the test for UV detection. Nonetheless, the variations in chain lengths associated with pH changes prevent us using R_2_S_2_ to determine the p*K*_a_ value of H_2_S_n_. The data also suggest that the R_2_S_2_ spectra of H_2_S_n_ depend on protonation as well as on the chain lengths (Fig. 2B). The chain length of H_2_S_n_ detected here should reflect the length in the solutions, as the method is optimized to ensure the derivatization reaction was fast enough to minimize chain elongation reactions [31]. However, if the derivatization step is low and if the alkylating agent reacts with the sulfane sulfur in the middle of polysulfides, some conversion reactions could occur, which interferes with the chain length detection [32].

### 3.3 RS_2_ of RS_n_R may correlate with the presence of thiosulfoxide

**Fig. 4:**
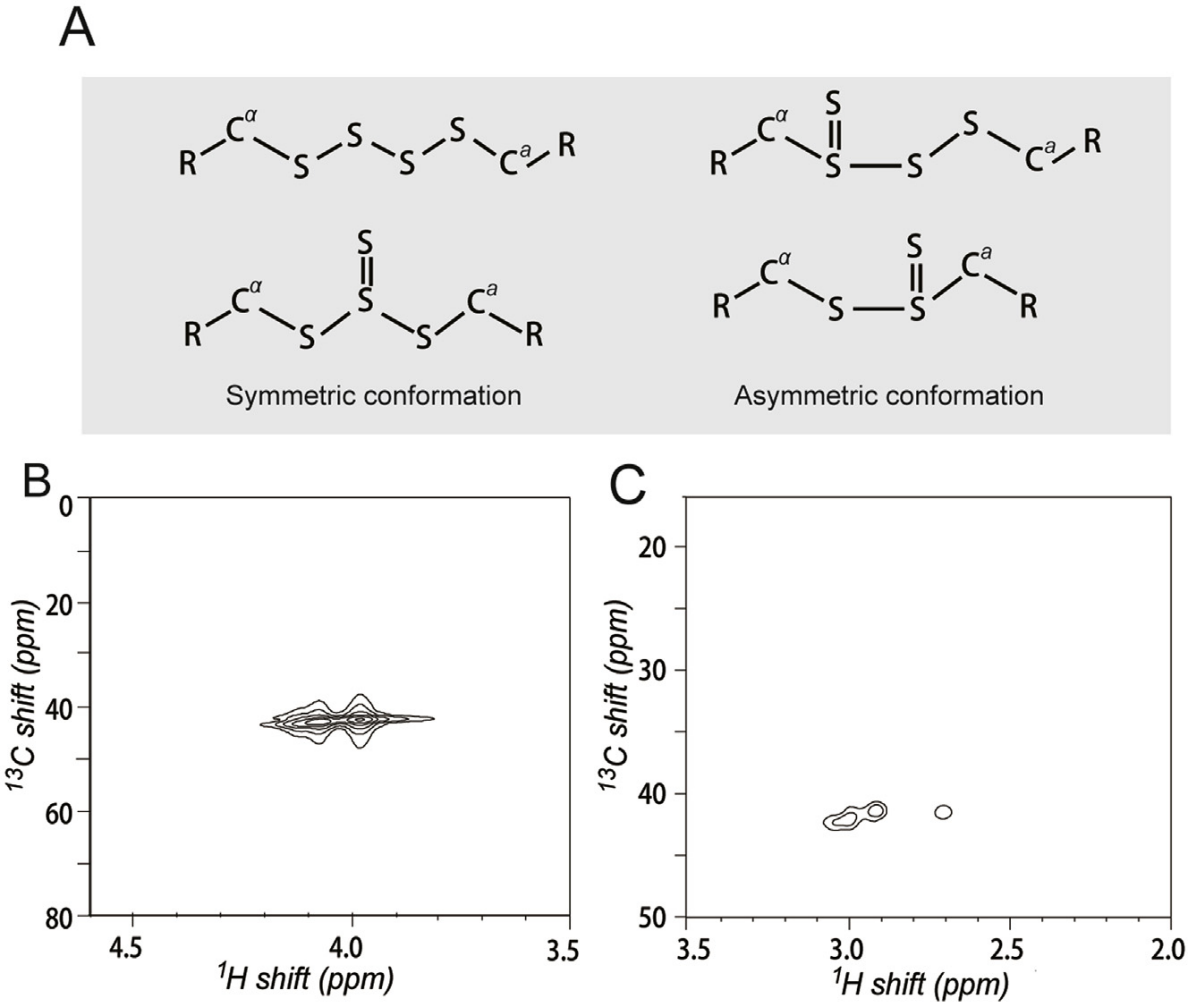
^**13**^**C–**^**1**^**H HMQC analysis of cluster 5 chemicals.** (A) Possible conformations of Pey-SSSS-Pey and Tsp-SSSS-Tsp. R is Pey or Tsp. Pey, prop-2-en-1-yl; Tsp, 3-(triethoxysilyl) prolyl. (B) ^13^C–^1^H HMQC spectra of Pey-SSSS-Pey. (C) ^13^C–^1^H HMQC spectra of Tsp-SSSS-Tsp. Data shown here are C^a^H_2_ and C^α^H_2_ spectra only; full spectra are provided in Figs. S4–S6.

RS_n_R contains sulfane sulfur that may tautomerize to a thiosulfoxide bond (sulfur-sulfur double bond, e.g., R_2_S = S) [33]. We hypothesized that RS_2_ of RS_n_R may correlate with the presence of thiosulfoxide. So we analyzed the structures of Pey-SSSS-Pey and Tsp-SSSS-Tsp by using ^1^H NMR and ^13^C–^1^H heteronuclear multiple quantum correlation spectroscopy (^13^C–^1^H HMQC). In ^13^C–^1^H HMQC spectra, the two -CH_2_- groups connecting to sulfur atoms in Pey-SSSS-Pey (C^a^ and C^α^) showed two distinguishable peaks, while those of Tsp-SSSS-Tsp showed three (Fig. 4, Fig. S4 and Fig. S5), suggesting C^a^ and C^α^ are not symmetrical. In ^1^H NMR spectra, protons linked to C^a^ and C^α^ had two or more groups of peaks, while those linked to other C's did not (Fig. S6 and Fig. S7). These results indicated the four sulfur atoms in these compounds are not linear and isomers containing the branched thiosulfoxide bond (>S=S) should exist, which causes the asymmetric configuration of C^a^ and C^α^. The branched thiosulfoxide bond might lead to the generation of sulfane sulfur, and the RS_2_ observed from cluster 5 chemicals might be caused by the presence of thiosulfoxide.

### 3.4 Analysis of reaction kinetics by using RS_2_

We used RS_2_ as a real-time probe in assays of reactive sulfane sulfur-involved reactions (Table 2). First, we tested the stability of RS_n_R chemicals (Me-SSS-Me and Tsp-SSSS-Tsp) in the presence of 100 mM GSH at pH 7.4 (100 mM HEPES). The RS_2_ spectra of RS_n_R were unchanged, and there was no H_2_S released from the solution. Thus, RS_n_R is rather stable. Second, we tested the reaction of H_2_S_n_ with GSH in deoxygenated HEPES buffer (100 mM, pH 7.4). After adding GSH (200 μM–5 mM) to 10 μM of H_2_S_n_, the RS_2_ spectra of H_2_S_n_ quickly decreased, and H_2_S was released. At low H_2_S_n_ concentrations and at pH 7.4, H_2_S_2_ is the dominant species [10]. By recording the RS_2_ decreases, we determined the 2^nd^-order rate constant of the reaction between H_2_S_n_ and GSH as 0.89 M^−1^s^−1^ (Table 2). Because GSH is at least two-orders-of-magnitude higher than H_2_S_n_ [14,29,34], the reaction between H_2_S_n_ and GSH should occur in the pseudo-first-order manner (e.g., t_1/2_ = 78 s) at the physiological pH and GSH concentration. Third, because RS_2_ has limitations, we could not use it to determine GSSH reduction at pH 7.4 due to its low RS_2_ signal.

**Table 2:** Kinetic analysis of reactive sulfane sulfur-involved reactions.

Reactions^a^	2^nd^-order rate constant (M^−1^s^−1^)
RS_n_R + DTT → DTT_oxdized_ + RS_n-1_R + H_2_S	0.52
H_2_S_n_ + GSH → GSSH + H_2_S	0.89
H_2_S_n_ + DTT → oxidized DTT + H_2_S	1.16
GSSH + H_2_O_2_ → GSSSG^b^ + 2H_2_O	23.76
2H_2_S + H_2_O_2_ → HSSH + 2OH^−^	0.46

a The reactions were conducted at 25 °C and pH 7.4.

b The GSSH preparation contains equal molar GSH; the reaction products are primarily GSSG and GSSSG [29].

Both H_2_S and GSSH were reported to have antioxidant functions, as evidenced by their reactivity towards H_2_O_2_ [14,16]. Using RS_2_ intensity curves of H_2_S_n_ and GSSSG concentration, we analyzed the kinetics of H_2_O_2_ with H_2_S or GSSH at pH 7.4 and 25 °C (Table 2). At pH 7.4 and 25 °C, H_2_S reacted with H_2_O_2_ slowly. The 2^nd^-order rate constant was determined to be 0.46 M^−1^s^−1^, close to a previously reported value (0.73 M^−1^s^−1^) determined at pH 7.4 and 37 °C [14]. On the other hand, GSSH rapidly reacted with H_2_O_2_ to produce GSSSG [29]; the 2^nd^-order rate constant was 23.8 M^−1^s^−1^ as determined with the RS_2_ increase of GSSSG, 50-fold higher than that between H_2_S and H_2_O_2_. The rate constant is likely an underestimate, as the GSSH preparation contains equal molar GSH; the reaction between H_2_O_2_ and GSSH/GSH primarily produces GSSG and GSSSG [29]. Considering GSSH is also more abundant than H_2_S inside cells, it has been proposed that GSSH is a major reactive oxygen species (ROS) scavenger other than H_2_S [29]; our finding proves the kinetic support for the hypothesis.

We also analyzed the reaction kinetics of GSSH with SSP4 at pH 7.4 and 25 °C by recording the fluorescence increase of the released chromophore from SSP4; the rate constant of this reaction was 9.53 M^−1^S^−1^.

### 3.5 Detection of GSSH disproportionation reactions

**Fig. 5:**
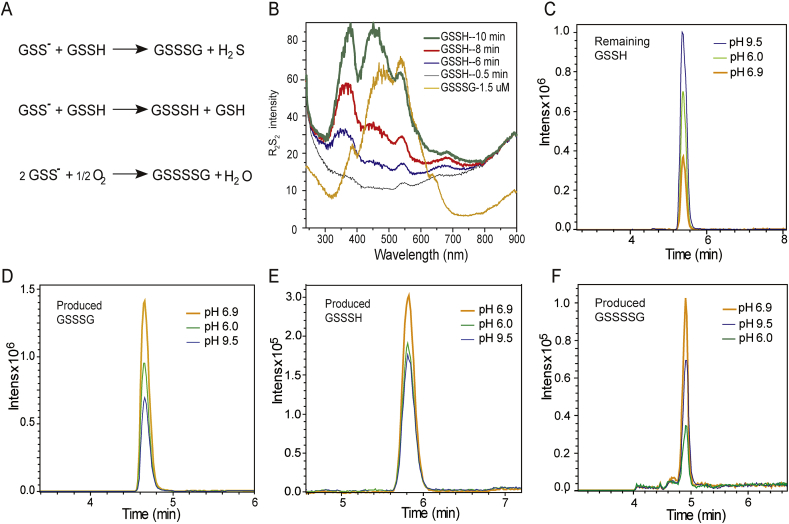
**RS**_**2**_**and LC-ESI-MS analysis of GSSH-involved chemical reactions.** (A) GSSH disproportionation and oxidation. (B) RS_2_ analysis of 50 μM GSSH disproportionation at pH 6.9. (C–F) LC-ESI-MS analysis of products from 50 μM GSSH disproportionation reactions. The reactions were performed at different pH for 10 min and derivatized with monobromobimane (mBBr) before LC-ESI-MS analysis.

Trace amounts of GSSSH and GSSSG have been found in cancer cells, whether they are from GSSH disproportionation reactions (Fig. 5A) are still inconclusive [29,35,36]. We studied these reactions using the RS_2_ method. When GSSH was incubated at pH 9.5, no appearance of RS_2_ was detected. At pH 6.9, RS_2_ spectra of protonated GSSH was initially observed, then it gradually changed to a spectrum overlapping those of GSSH and GSSSG (Fig. 5B). At pH 6.0, the RS_2_ spectral change was also observed with a slower increase of the GSSSG peak. In consistent, LC-ESI-MS analysis (Fig. S8) indicated the amount of unreacted GSSH (remaining in solution) was the highest at pH 9.5 and the lowest at pH 6.9 (Fig. 5C). GSSSG was produced the most at pH 6.9 with less at pH 6.0 and the lowest at pH 9.5 (Fig. 5D). GSSSH was also produced, but at about one order of magnitude lower than that of GSSSG, following the same trend at various pH values (Fig. 5E; pH 6.9 > pH 6.0 > pH 9.5). At pH 9.5, a small amount of GSSSSG was also detected, which should be produced from GSS^−^ oxidation (Fig. 5F). These results indicated that GSSH disproportionation occurred most efficiently at its p*K*_a_. Considering GSSH can be as high as 100 μM in cancer cell and its p*K*_a_ is close to the intracellular pH [29], it is highly possible that the intracellular GSSSH and GSSSG are produced from these reactions.

### 3.6 RS_2_ separates protein S-persulfidation into active and inactive forms

**Fig. 6:**
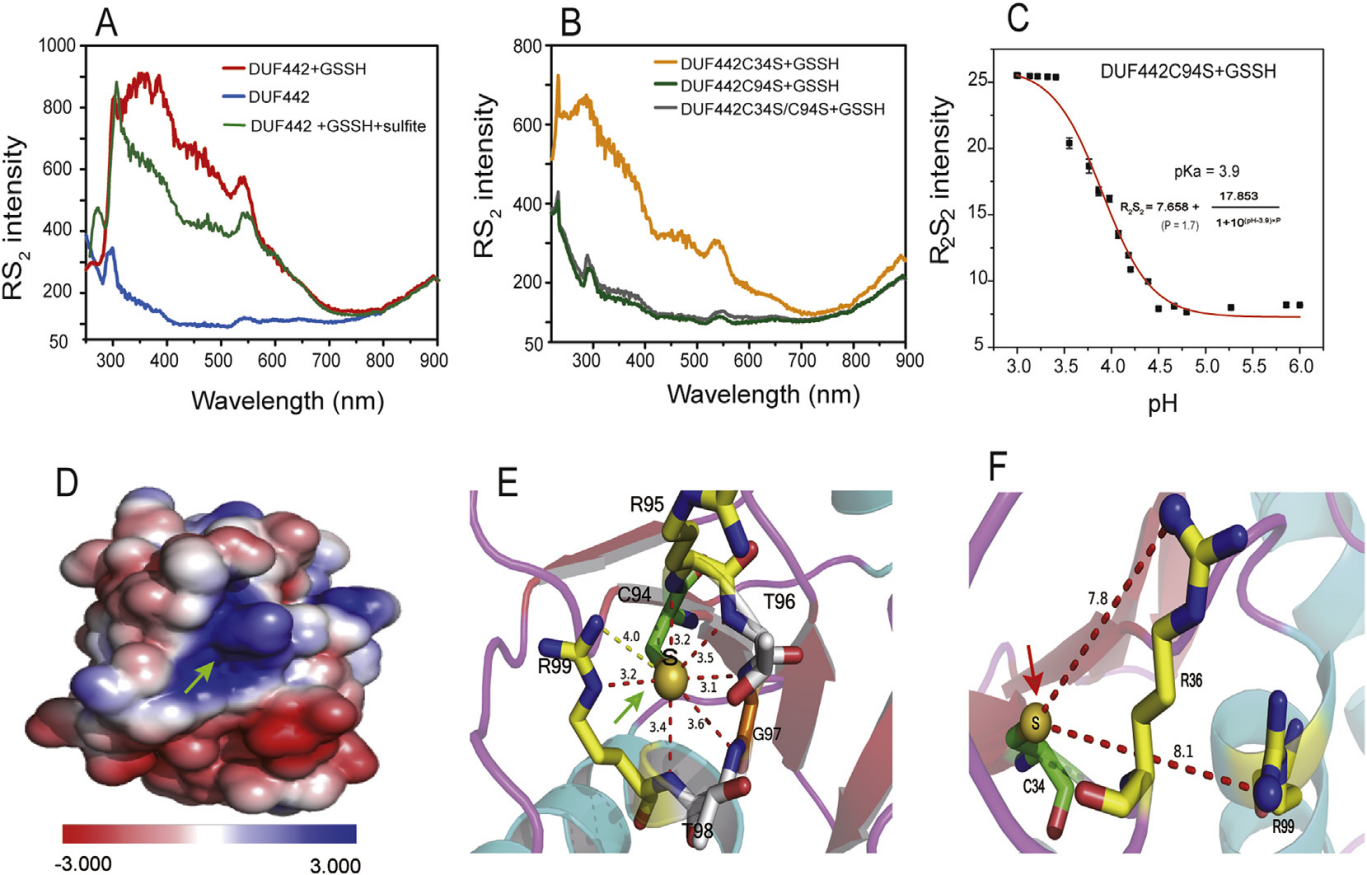
**The persulfide at the active site of DUF442 (C94) is protonated in a positively charged pocket.** (A) RS_2_ analysis of DUF442, DUF442 persulfide, or after the treatment of DUF442 persulfide with sulfite. (B) RS_2_ analysis of DUF442 mutant persulfides. (C) R_2_S_2_ analysis of DUF442C34-SSH with the DUF442C94S mutant at different pH. The data was used to estimate the p*K*_a_ of DUF442C34-SSH. (D, E, F) Modeled 3D structure of DUF442. The green arrow points to the sulfur atom of C94, which locates at the bottom of a positive electrostatic field pocket and is surrounded by –NH_2_– and –NH_3_^+^ groups (D and E). The red arrow points to the sulfur atom of C34, which is > 7.8 Å away from the nearest –NH_3_^+^ group (F). The distances (Å) from the sulfur atom to the circumjacent nitrogen atoms of the peptide backbone (red dotted line) and the side chain of R99 (yellow dotted line) were shown.

DUF442, a domain of *Cupriavidus pinatubonensis* JMP134 sulfide:quionone oxidoreductase (GeneBank: AAZ62946.1), has rhodanese activity and catalyzes the reaction of GSSH with sulfite to produce thiosulfate [10]. The DUF442 domain consists of 128 amino acid residues with two cysteine residues, C34 and C94, and only C94 is conserved and functionally essential [10]. We used GSSH to react with DUF442 and LC-MS/MS to analyze the modification. Both C34-SSH and C94-SSH modifications were detected (Fig. S9 and Fig. S10). The modified DUF442 displayed significant RS_2_, which was not observed from unmodified protein at pH 7.4 (Fig. 6A). In addition, the C34S/C94S double-mutant DUF442 showed no RS_2_ after reacting with GSSH (Fig. 6B). Next, we reacted GSSH with the two single-mutants of DUF442 (C34S and C94S) at pH 7.4. C34S mutant showed significant RS_2_, while C94S mutant did not, although the individual Cys residues were modified by GSSH treatment to form persulfides (confirmed via LC-MS/MS analysis). These results indicated that C94-SSH is likely in the protonated form (C94-SSH) and C34-SSH is in the deprotonated form (C34-SS^-^) at pH 7.4. When reacted sulfite, the RS_2_ intensity of C94-SSH (C34S mutant) significantly decreased with the production of thiosulfate (C94-SSH + SO_3_^2−^ →C94-SH + S_2_O_3_^2−^) [10]; whereas, C34-SSH (C94S mutant) without RS_2_ did not produce thiosulfate when reacted with sulfite. When C34-SSH was titrated with HCl, the RS_2_ intensity was increased at low pH. In the control containing C34-SH (GSSH unreacted), RS_2_ was not detectable at all the tested pH. Considering HCl titration may cause aggregation of protein, which disturbs RS_2_ measuring, we used different pH buffer for the titration. We diluted C34-SH or C34-SSH protein in HEPES buffers of different pH. The p*K*_a_ was determined by using R_2_S_2_ method to be 6.29 (Fig. 6C). Thus, only C94-SSH in the DUF442 wild type or the DUF442C34S mutant is protonated at pH 7.4 and the sulfane sulfur can be transferred to sulfite to produce thiosulfate.

To inspect what makes DUF442-C94-SSH in the protonated form (C94-SSH) at pH 7.4, we modeled 3D structures of DUF442 with a putative rhodanese from *Neisseria meningitides* z2491 (PDB ID: 2F46) as the template (39% sequence similarity). The C94 sulfur was located at the bottom of a cradle-like pocket surrounded by basic side chains, generating a positively electrostatic field (Fig. 6D). The distances between the sulfur atom and the circumjacent nitrogen atoms of the peptide backbone are in the range of 3.1 Å—4.0 Å, and the NH_3_^+^ group of R99 is also nearby (Fig. 6E). Therefore, either C94-SSH is not dissociated in the pocket or C94-SS- forms a hydrogen bond with one of these groups as revealed by RS_2_. In contrast, sulfur atom of C34 is not located in a positively electrostatic field (Fig. 6F), and it should exist in the deprotonated form C34-SS- at pH 7.4, which showed no RS_2_ and electrophilicity.

### 3.7 RS_2_ method application in whole cells

**Fig. 7:**
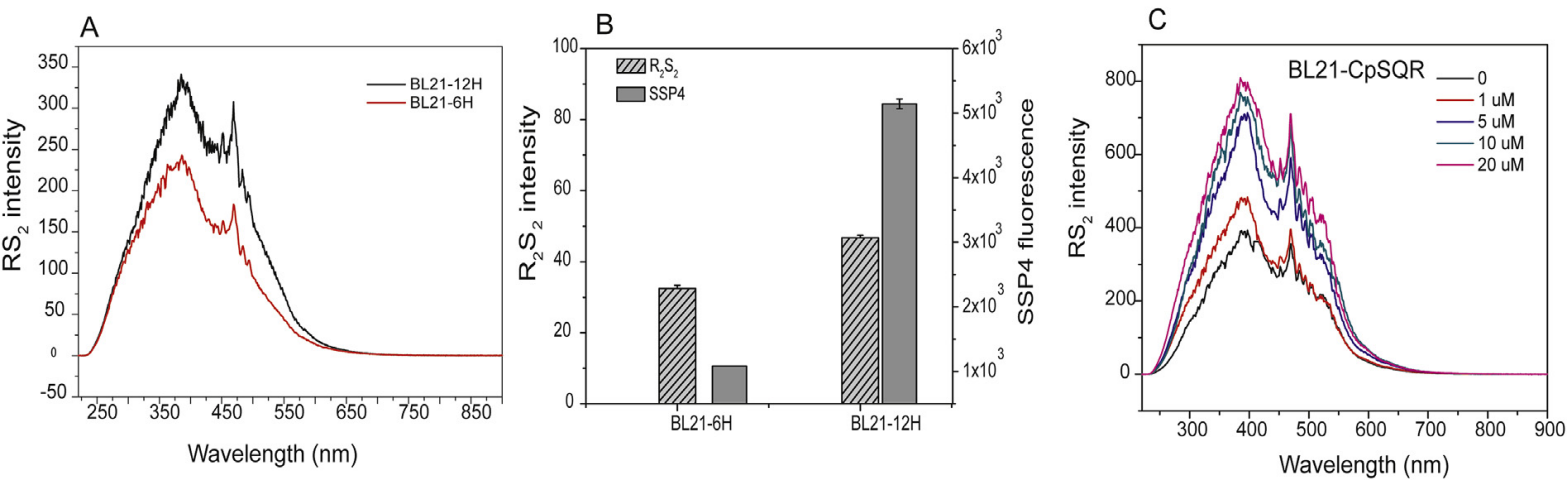
**RS**_**2**_**analysis of reactive sulfane sulfur in *E. coli* cells.** (A) RS_2_ analysis of intracellular **reactive sulfane sulfur** in wild type *E. coli* at different growth phases in LB medium. (B) Reactive sulfane sulfur analysis of *E. coli* cells by using R_2_S_2_ and SSP4 at 6 h or 12 h of incubation. (C) RS_2_ analysis of recombinant *E. coli* harboring CpSQR after the cells oxidized different amount of H_2_S.

We also used the RS_2_ method to analyze intracellular changes of reactive sulfane sulfur in wild-type *E. coli*. *E. coli* contained more reactive sulfane sulfur at the stationary phase of growth (12 h) than at the log phase (6 h), as revealed by RS_2_ intensity (Fig. 7A) and SSP4 analysis (Fig. 7B). The sulfane sulfur species have different RS_2_ spectra at pH 7.4 (Fig. 1, Fig. 5, Fig. 6A). The RS_2_ peak of whole cells around 400 nm suggest the presence of R-SS_n_H and R-SS_n_R (n ≥ 2), as well as persulfides (R-SSH); however, most protein persulfides and GSSH do not contribute much to the RS_2_ signal at the physiological pH. The low intensity of persulfides at physiological pH is likely responsible for a smaller increase in the R_2_S_2_ signal than the increase of sulfane sulfur detected by SSP4 for cells at the stationary phase (Fig. 7B). Further, the RS_2_ peak of whole cells at 450 nm suggest the possible presence of H_2_S_n_.

Previously, we reported that recombinant *E. coli* strain expressing a sulfide:quinone oxidoreductase of *Cupriavidus pinatubonensis* JMP134 (CpSQR) can oxidize H_2_S to H_2_S_n_, and the produced H_2_S_n_ is associated with the cell [10]. Herein, we used the recombinant *E. coli* to oxidize H_2_S. After the cells oxidized H_2_S, the cells were harvested, washed, and diluted for RS_2_ measurement. The RS_2_ peak at 450 nm increased, suggesting the production of H_2_S_n_; however, the increase slowed down with increased H_2_S oxidation (Fig. 7C). On the basis of the RS_2_ spectra, other reactive sulfane sulfur species inside *E. coli* also increased after H_2_S oxidation, possibly including protein-SSH.

## 4 Discussion

Individual fluorophores are often considered as simultaneous photon absorbers and emitters with no significant light scattering due to small sizes [37]. Consequently, RS_2_ is often observed from the aggregated fluorophores, which usually are simultaneous photon absorbers, scatters, and fluorescence emitters [21]. However, RS_2_ of reactive sulfane sulfur is most likely from soluble, individual molecules, since GSSH at pH 6 and DUF442-C94S-SSH at pH 7.4 are soluble and have RS_2_ (Fig. 2, Fig. 6). At pH 8 to 8.5, H_2_S_n_ is mainly present as S_2_^2−^ (Fig. S3) and the solution has low RS_2_ (Fig. 2B). At pH > 9, long chain S_n_^2−^ species are dominant and RS_2_ was increased (Fig. 2B and Fig. S3). Our data showed that GSSH in the protonated form has RS_2_ and is electrophilic, and the deprotonated form does not have RS_2_ and is not electrophilic (Fig. 3A&B). The ratio of GSSH/GSS^−^ at various pH determines the electrophilicity and RS_2_ intensity (Fig. 3D). Apparently, the electrophilicity of the sulfane sulfur in GSSH is strongly affected by deprotonation because the negatively charged terminal sulfur (S^−^) affects its adjacent sulfur through the α-effect, making both sulfur atoms with negative charge. The same logic may also apply to long chain S_n_^2−^ (n > 4) with the terminal sulfur (S^−^) having minimal effects on distant S in the middle (Fig. S3). The electrophilicity of sulfane sulfur can be explained in the form of thiosulfoxide (RR’S=S) [33,38]. Although our NMR analysis showed evidence to support the presence of thiosulfoxide in R-SSSS-R (Fig. 4), for GSSH and H_2_S_n_, whether the sulfane sulfur is in present as thiosulfoxide or in a linear form is still unsettled [39,40]. Thus, our results associate RS_2_ with the electrophilicity of sulfane sulfur; the deprotonated persulfides (R-SS^-^) are nucleophilic and prone to oxidation but does not react with SSP4 [41].

The p*K*_a_ values of thiols are critical to their reactivity at physiological pH. The p*K*_a_ of Cys thiol at active center of enzyme may be lowered so that the thiol is deprotonated at neutral pH, which are strongly nucleophilic and are prone to oxidation by ROS. The p*K*_a_ values of R-SSH are also likely important and have previously been reported within the range of 4.3–6.23 [16,42], implying that the persulfides should be mostly in the deprotonated form (RSS^-^) at pH 7 and displaying nucleophilic properties. The p*K*_a_ value of cumyl-SSH has recently being determined as 7.0 [43], close to the value of GSSH (6.9) that we determined with two different approaches (Fig. 3). According to this value, the ratio of deprotonated form and protonated form of GSSH is within the range of 2–9 at physiological pH range (7.2–7.8). Disproportionation of GSSH requires both the deprotonated and protonated forms with one playing an electrophile and another acting as a nucleophile, which is consistent with our observation that the disproportionation was the most efficient at pH closed to its p*K*_a_ (Fig. 3). These chemical reactions might be the origin of intracellular GSSSH and GSSSG. Sulfane sulfur prefers to move from a high reactive polysulfide to form a lower one [10,44]. Thus, in the cell the flow of sulfane sulfur is likely from H_2_S_n_ to GSSH and then to GSSSG.

Protein S-persulfidation is common inside cells [39]. Here we showed that like cystinyl thiols at the active site, the p*K*_a_ values of protein persulfides can also be affected by its location. Most protein persulfides are likely deprotonated at physiological pH because they have no apparent RS_2_ and cannot react with sulfite (Fig. 6), but the sulfane sulfur at the active site of rhodanese is not deprotonated, due to its location in a positive electrostatic field. Rhodanese can then transfer the sulfane sulfur to small nucleophiles, such as cyanide and sulfite, which act as sulfane sulfur acceptors. Our finding implies that the catalysis of rhodanese is likely to generate an electrophilic sulfane sulfur that is easily transferred between two nucleophilic substrates, such as from GSS^−^ to SO_3_^2−^, producing S_2_O_3_^2−^.

## 5 Conclusions

We discovered reactive sulfane sulfur species have RS_2_ properties only when the molecules contain an electrophilic sulfane sulfur. It can be applied to reactive sulfane sulfur analyses, such as p*K*_a_ determination, reaction kinetics, pH-dependent sulfane reactivity of small and protein persulfides, etc. For whole cell analysis, it may reveal the relative abundance of a reactive sulfane sulfur species. The RS_2_ method is rapid, sensitive and convenient, allowing us to reveal several new chemical and biochemical properties of biologically relevant reactive sulfane sulfur. The results that were reported here, such as the p*K*_a_ of GSSH, the reaction parameters, the distribution of H_2_S_n_ species at different pH, may fill some gaps in the field.

## Conflicts of interest

The authors declare no conflicts of interest.

## Funding

The work was financially supported by grants from the 10.13039/501100001809National Natural Science Foundation of China (91751207, 31770093), the 10.13039/501100012166National Key Research and Development Program of China (2016YFA0601103), and the 10.13039/501100007129Natural Science Foundation of Shandong Province, China (ZR2016CM03, ZR2017ZB0210).

## References

[1] Kolluru G.K., Shen X., Kevil C.G. (2013). A tale of two gases: NO and H_2_S, foes or friends for life?. Redox Biol..

[2] Olson K.R. (2005). Vascular actions of hydrogen sulfide in nonmammalian vertebrates. Antioxidants Redox Signal..

[3] Rajpal S., Katikaneni P., Deshotels M., Pardue S., Glawe J. (2018). Total sulfane sulfur bioavailability reflects ethnic and gender disparities in cardiovascular disease. Redox Biol..

[4] Greiner R., Pálinkás Z., Bäsell K., Becher D., Antelmann H. (2013). Polysulfides link H_2_S to protein thiol oxidation. Antioxidants Redox Signal..

[5] Liu H., Radford M.N., Yang C.T., Chen W., Xian M. (2019). Inorganic hydrogen polysulfides: chemistry, chemical biology and detection. Br. J. Pharmacol..

[6] Huang Y., Yu F.B., Wang J.C., Chen L.X. (2016). Near-Infrared fluorescence probe for in situ detection of superoxide anion and hydrogen polysulfides in mitochondrial oxidative stress. Anal. Chem..

[7] Bibli S.I., Luck B., Zukunft S., Wittig J., Chen W. (2018). A selective and sensitive method for quantification of endogenous polysulfide production in biological samples. Redox Biol..

[8] Ono K., Akaike T., Sawa T., Kumagai Y., Wink D.A. (2014). The redox chemistry and chemical biology of H_2_S, hydropersulfides and derived species: implications to their possible biological activity and utility. Free Radical Biol. Med..

[9] Olson K.R., Gao Y., Deleon E.R., Arif M., Arif F. (2017). Catalase as a sulfide-sulfur oxido-reductase: an ancient (and modern?) regulator of reactive sulfur species (RSS). Redox Biol..

[10] Xin Y., Liu H., Cui F., Xun L. (2016). Recombinant *Escherichia coli* with sulfide: quinone oxidoreductase and persulfide dioxygenase rapidly oxidizes sulfide to sulfite and thiosulfate via a new pathway. Environ. Microbiol..

[11] Akaike T., Ida T., Wei F.Y., Nishida M., Kumagai Y. (2017). Cysteinyl-tRNA synthetase governs cysteine polysulfidation and mitochondrial bioenergetics. Nat. Commun..

[12] Toohey J.I. (2011). Sulfur signaling: is the agent sulfide or sulfane?. Anal. Biochem..

[13] Cerda M.M., Pluth M.D. (2018). S marks the spot: linking the antioxidant activity of N-Acetyl cysteine to H_2_S and sulfane sulfur species. Cell Chem. Biol..

[14] Mishanina T.V., Libiad M., Banerjee R. (2015). Biogenesis of reactive sulfur species for signaling by hydrogen sulfide oxidation pathways. Nat. Chem. Biol..

[15] Libiad M., Yadav P.K., Vitvitsky V., Martinov M., Banerjee R. (2014). Organization of the human mitochondrial hydrogen sulfide oxidation pathway. J. Biol. Chem..

[16] Mustafa A.K., Gadalla M.M., Sen N., Kim S., Mu W. (2009). H_2_S signals through protein s-sulfhydration. Sci. Signal..

[17] Tang T., Li X., Liu X., Wang Y.L., Ji C.C. (2018). A single-domain rhodanese homologue MnRDH1 helps to maintain redox balance in &ITMacrobrachium nipponense&IT. Dev. Comp. Immunol..

[18] Bordo D., Bork P. (2002). The rhodanese/Cdc 25 phosphatase superfamily-Sequence-structure-function relations. EMBO Rep..

[19] Gao M., Yu F.B., Chen H., Chen L.X. (2015). Near-infrared fluorescent probe for imaging mitochondrial hydrogen polysulfides in living cells and in vivo. Anal. Chem..

[20] Takano Y., Hanaoka K., Shimamoto K., Miyamoto R., Komatsu T. (2017). Development of a reversible fluorescent probe for reactive sulfur species, sulfane sulfur, and its biological application. Chem. Commun..

[21] Gao M., Wang R., Yu F.B., Chen L.X. (2018). Evaluation of sulfane sulfur bioeffects via a mitochondria-targeting selenium-containing near-infrared fluorescent probe. Biomaterials.

[22] Gao M., Wang R., Yu F.B., Li B.W., Chen L.X. (2018). Imaging of intracellular sulfane sulfur expression changes under hypoxic stress via a selenium-containing near-infrared fluorescent probe. J. Mater. Chem. B.

[23] Gao M., Wang R., Yu F.B., Youc J.M., Chen L.X. (2018). Imaging and evaluation of sulfane sulfur in acute brain ischemia using a mitochondria-targeted near-infrared fluorescent probe. J. Mater. Chem. B.

[24] Lloyd J.B.F., Evett I.W. (1977). Prediction of peak wavelengths and intensities in synchronously excited fluorescence emission-spectra. Anal. Chem..

[25] Moutiez M., Aumercier M., Teissier E., Parmentier B., Tartar A. (1994). Reduction of a trisulfide derivative of glutathione by glutathione reductase. Biochem. Biophys. Res. Commun..

[26] Luebke J.L., Shen J., Bruce K.E., Kehl-Fie T.E., Peng H. (2014). The CsoR-like sulfurtransferase repressor (CstR) is a persulfide sensor in *Staphylococcus aureus*. Mol. Microbiol..

[27] Nettles C.B., Zhou Y.D., Zou S.L., Zhang D.M. (2016). UV-Vis ratiometric resonance synchronous spectroscopy for determination of nanoparticle and molecular optical cross sections. Anal. Chem..

[28] Bax A., Griffey R.H., Hawkins B.L. (1983). Correlation of proton and nitrogen-15 chemical shifts by multiple quantum NMR. J. Magn. Reson..

[29] Ida T., Sawa T., Ihara H., Tsuchiya Y., Watanabe Y. (2014). Reactive cysteine persulfides and S-polythiolation regulate oxidative stress and redox signaling. Proc. Natl. Acad. Sci. U. S. A.

[30] Xia Y., Chu W., Qi Q., Xun L. (2015). New insights into the QuikChangeTM process guide the use of Phusion DNA polymerase for site-directed mutagenesis. Nucleic Acids Res..

[31] Alexey Kamyshny J., Goifman A., Gun J., Rizkov Dan, Lev O. (2004). Equilibrium distribution of polysulfide Ions in aqueous solutions at 25°C:  A new approach for the study of polysulfides' equilibria. Environ. Sci. Technol..

[32] Bogdandi V., Ida T., Sutton T.R., Bianco C., Ditroi T. (2019). Speciation of reactive sulfur species and their reactions with alkylating agents: do we have any clue about what is present inside the cell?. Br. J. Pharmacol..

[33] Kutney G.W., Turnbull K. (1982). Compounds containing the sulfur-sulfur double bond. Chem. Rev..

[34] Kimura H. (2015). Signaling molecules: hydrogen sulfide and polysulfide. Antioxidants Redox Signal..

[35] Toohey J.I., Cooper A.J.L. (2014). Thiosulfoxide (Sulfane) sulfur: new chemistry and new regulatory roles in biology. Molecules.

[36] Chen W., Liu C., Peng B., Zhao Y., Pacheco A. (2013). New fluorescent probes for sulfane sulfurs and the application in bioimaging. Chem. Sci..

[37] Vithanage B.C.N., Xu J.N.X.Z., Zhang D.M. (2018). Optical properties and kinetics: new insights to the porphyrin assembly and disassembly by polarized resonance synchronous spectroscopy. J. Phys. Chem. B.

[38] Toohey J.I., Cooper A.J.L. (2014). Thiosulfoxide (Sulfane) sulfur: new chemistry and new regulatory roles in biology. Molecules.

[39] Park C.M., Weerasinghe L., Day J.J., Fukuto J.M., Xian M. (2015). Persulfides: current knowledge and challenges in chemistry and chemical biology. Mol. Biosyst..

[40] Steudel R., Drozdova Y., Miaskiewicz K., Hertwig R.H., Koch W. (1997). How unstable are thiosulfoxides? An ab initio MO study of various disulfanes RSSR (R=H, Me, Pr, All), their branched isomers R(2)SS, and the related transition states. J. Am. Chem. Soc..

[41] Cuevasanta E., Lange M., Bonanata J., Coitino E.L., Ferrer-Sueta G. (2015). Reaction of hydrogen sulfide with disulfide and sulfenic acid to form the strongly nucleophilic persulfide. J. Biol. Chem..

[42] Everett S.A., Folkes L.K., Wardman P., Asmus K.D. (1994). Free-radical repair by a novel perthiol: reversible hydrogen transfer and perthiyl radical formation. Free Radic. Res..

[43] Chauvin J.P.R., Griesser M., Pratt D.A. (2017). Hydropersulfides: H-atom transfer agents par excellence. J. Am. Chem. Soc..

[44] Melideo S.L., Jackson M.R., Jorns M.S. (2014). Biosynthesis of a central Intermediate in hydrogen sulfide metabolism by a novel human sulfurtransferase and its yeast ortholog. Biochemistry.

