# Supplementary material for: Using resonance synchronous spectroscopy to characterize the reactivity and electrophilicity of biologically relevant sulfane sulfur
Source: Redox Biol. 2019 Mar 26;24:101179. doi: 10.1016/j.redox.2019.101179 (PMC6441731; doi:10.1016/j.redox.2019.101179)
Supplement: Multimedia component 1 [file mmc1.pdf]

Supplementary Table 1 and Supplementary Figures 1-10

Using resonance synchronous spectroscopy to characterize the reactivity and electrophilicity of biologically relevant sulfane sulfur

Huanjie Li[a], Huaiwei Liu[a,*], Zhigang Chen[a], Rui Zhao[a], Qingda Wang[a], Mingxue Ran[a], Yongzhen Xia[a], Xin Hu[b], Jihua Liu[b], Ming Xian[c], Luying Xun[a,d,*]

Table S1. Strains and plasmids used in this study

Strain/plasmid	Characteristic	Source
Escherichia coli strain		
DH5α	F^- *endA1, glnV44, thi-1, recA1, relA1, gyrA96, deoR, nupG, purB20,* φ80d,*lacZ,*ΔM15, Δ(*lacZYA-argF*), U169, hsdR17($r_K^- m_K^+$), λ^-	Invitrogen
BL21(DE3)	*E. coli* str. B F^-, *ompT, gal, dcm lon ,hsdS*$_B$($r_B^- m_B^-$) λ(DE3 [*lacI lacUV5-T7p07 ind1 sam7 nin5*]) , [$malB^+$]$_{K\text{-}12}$(λ^S)	Invitrogen
DH5α	Cloning strain	Invitrogen
BL21(DE3)	Protein expression strain	Invitrogen
Plasimd		
pET30a-DUF442	pET30a containing the DUF442 domain of CpSQR from *C. pinatubonensis* JMP134 with C-terminal His-tag (GeneBank: AAZ62946.1).	In the lab
pET30a-DUF442$_{C34S}$	pET30a containing the DUF442 domain with C-terminal His-tag and the C34S mutation.	This study

pET30a-DUF442$_{C94S}$	pET30a containing the DUF442 domain with C-terminal His-tag and the C94S mutation	This study
pET30a-DUF442$_{C34S/C94S}$	pET30a containing the DUF442 domain with C-terminal His-tag and the C34S and C94S double mutation	This study
pBBR1-SQR	pBBR1 containing the CpSQR from *C. pinatubonensis* JMP134 (GeneBank: AAZ62946.1).	This study

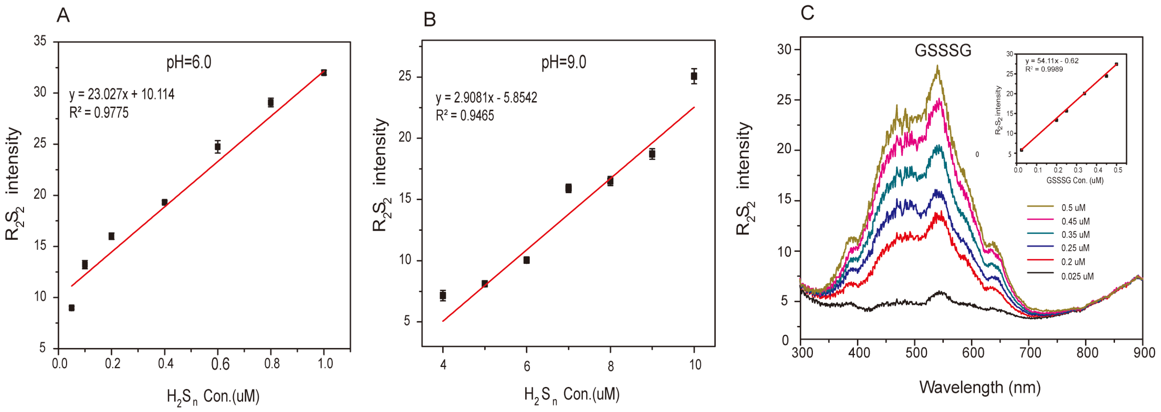

Figure S1. R_2S_2 analysis of H_2S_n and GSSSG. (A) & (B) H_2S_n diluted in Tris-HCl buffer (50 mM, pH 6.0 and 9.0) displays concentration-dependent, linear responses of R_2S_2. **(C)** GSSSG showed good responses between its concentrations and R_2S_2 intensity

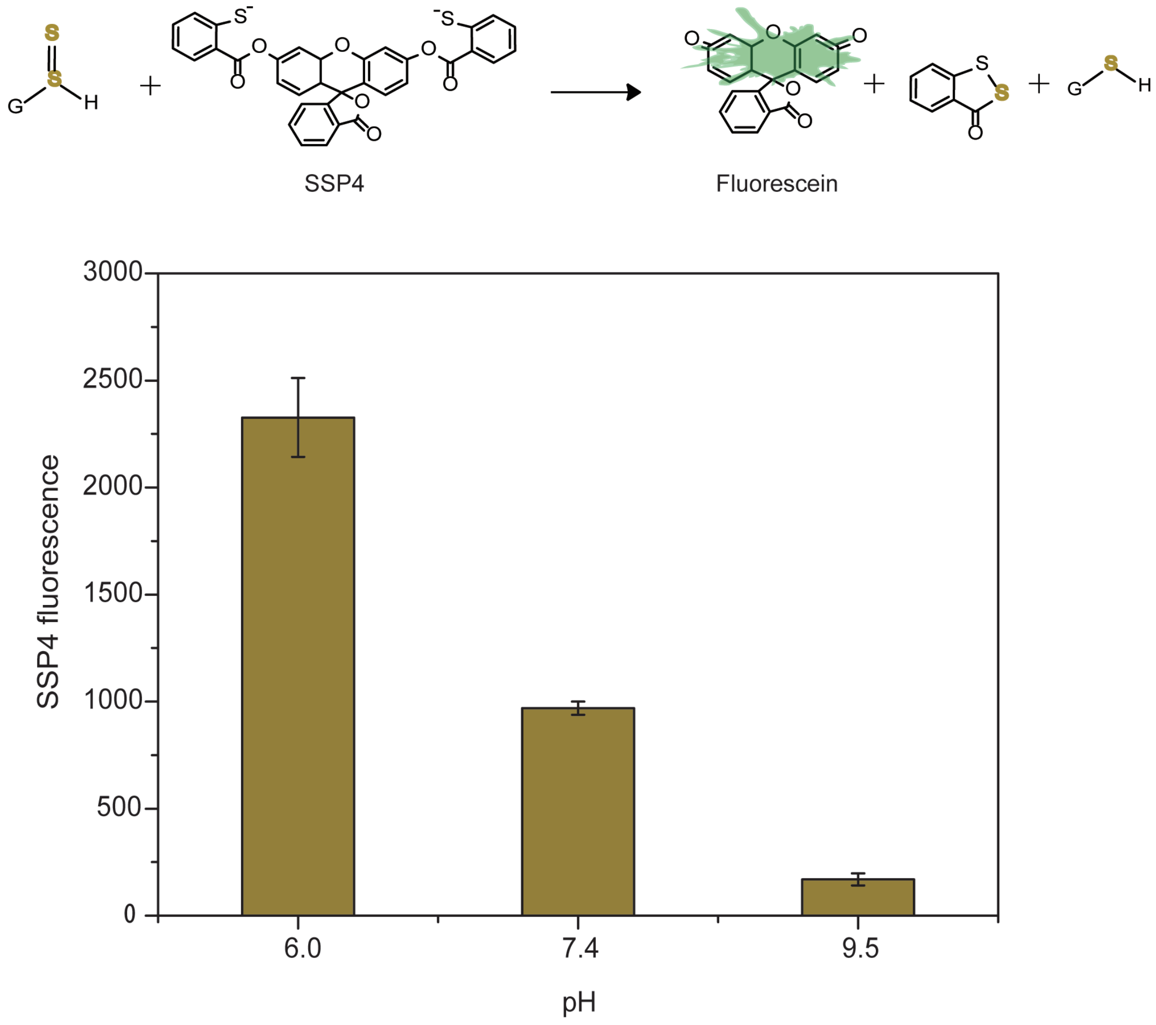

Fig. S2. Reaction of SSP4 with GSSH at different pH range. GSSH reacted with non-fluorescent sulfane sulphur probe 4 (SSP4) to release fluorescent fluorescein. When 20 μM GSSH and 10 μM SSP4 were mixed at different pH for 15 min, the reaction was rapid at pH 6 but not at pH 9.5.

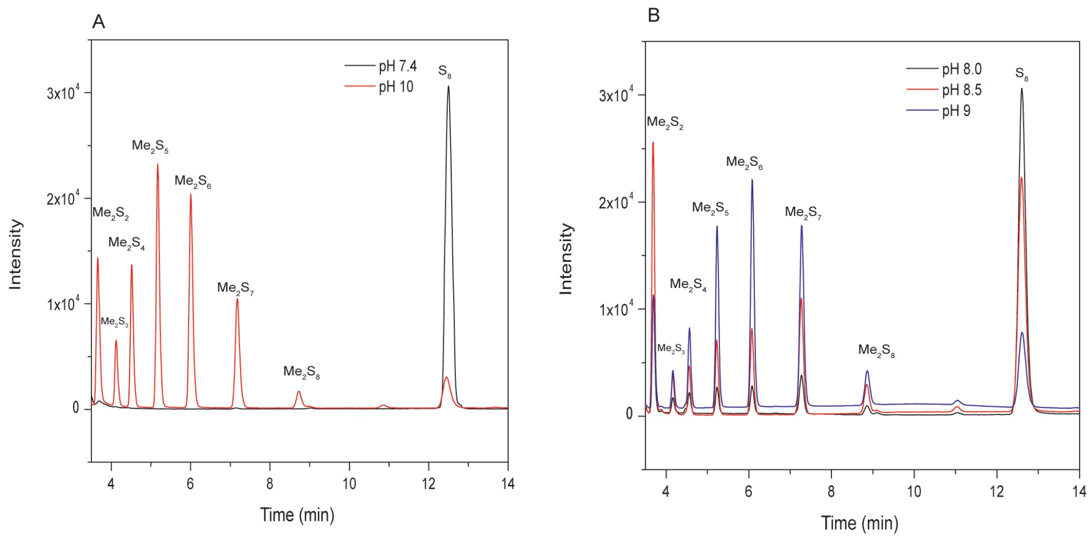

Fig. S3. HPLC analysis of 5 mM H_2S_n diluted in Tris-HCl buffer at different pH. Typical chromatograms of dimethylpolysulfides obtained by derivatization of H_2S_n with methyl trifluoromethanesulfonate. (A) H_2S_n diluted in Tris-HCl buffer at pH 7.4 and 10; (B) H_2S_n diluted in Tris-HCl buffer at pH8.0, 8.5, and 9. The samples were detected with UV absorption (254 nm).

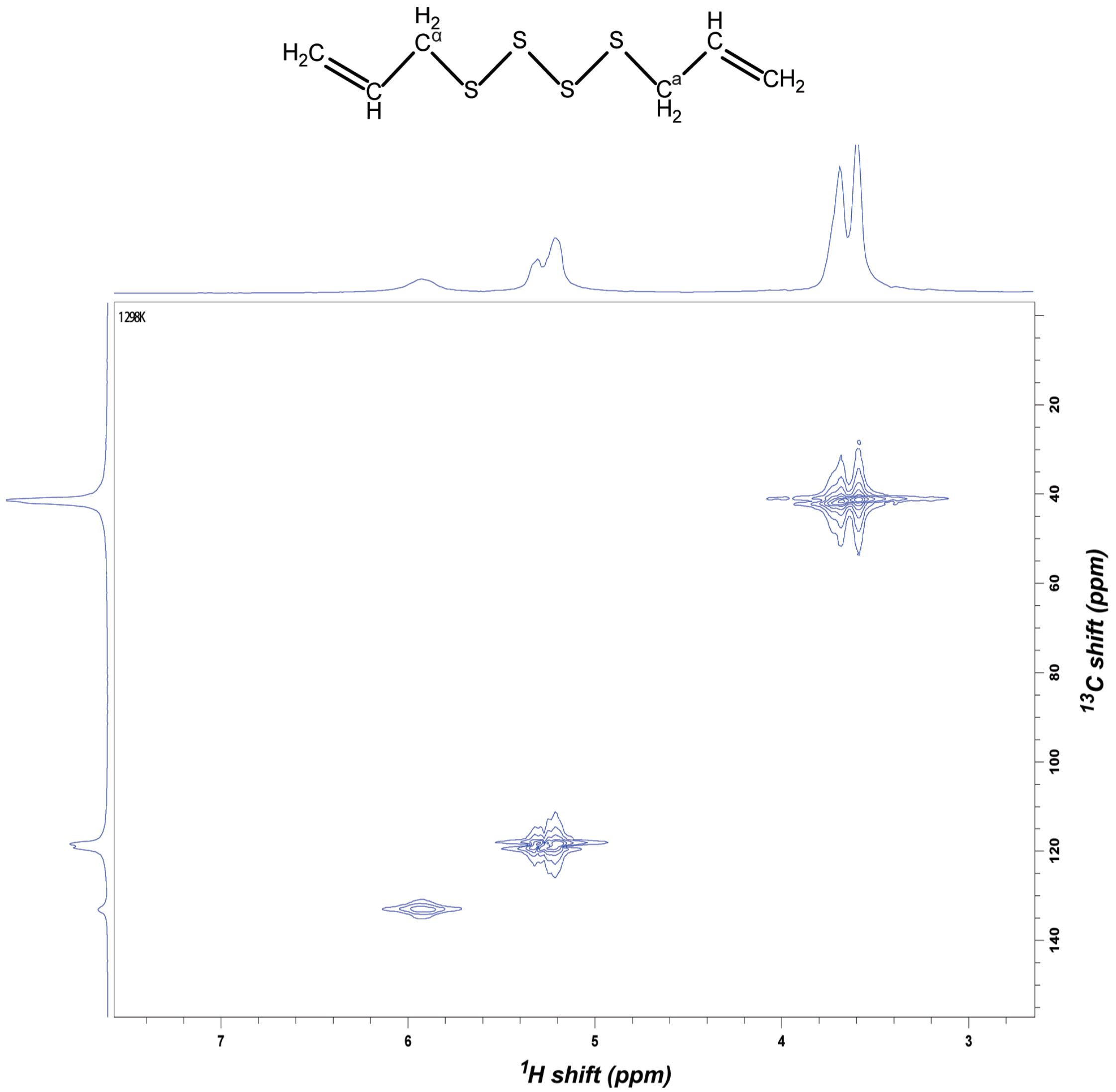

Fig. S4. **^{13}C–^{1}H HMQC spectra of Pey-SSSS-Pey. From down-left to up-right: –CH=, =CH_2, –$C^{\alpha}H_2$–(S) and –$C^{a}H_2$–(S).**

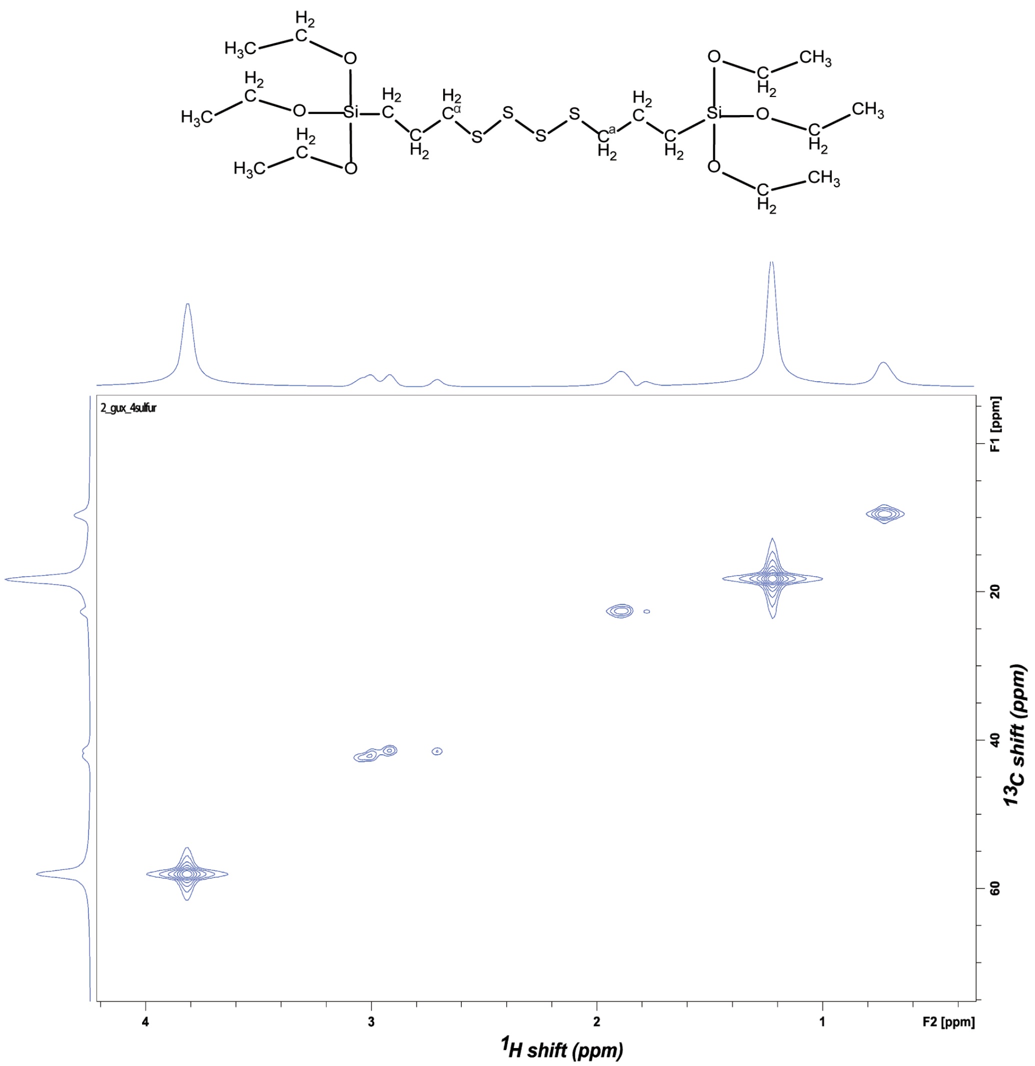

Fig. S5. ^{13}C–^{1}H **HMQC spectra of Tsp-SSSS-Tsp. From down-left to up-right: –CH₂–(O), –C$^{\alpha}$H₂–(S) and –C^{a}H₂–(S), –CH₂–, –CH₃, –CH₂–(Si).**

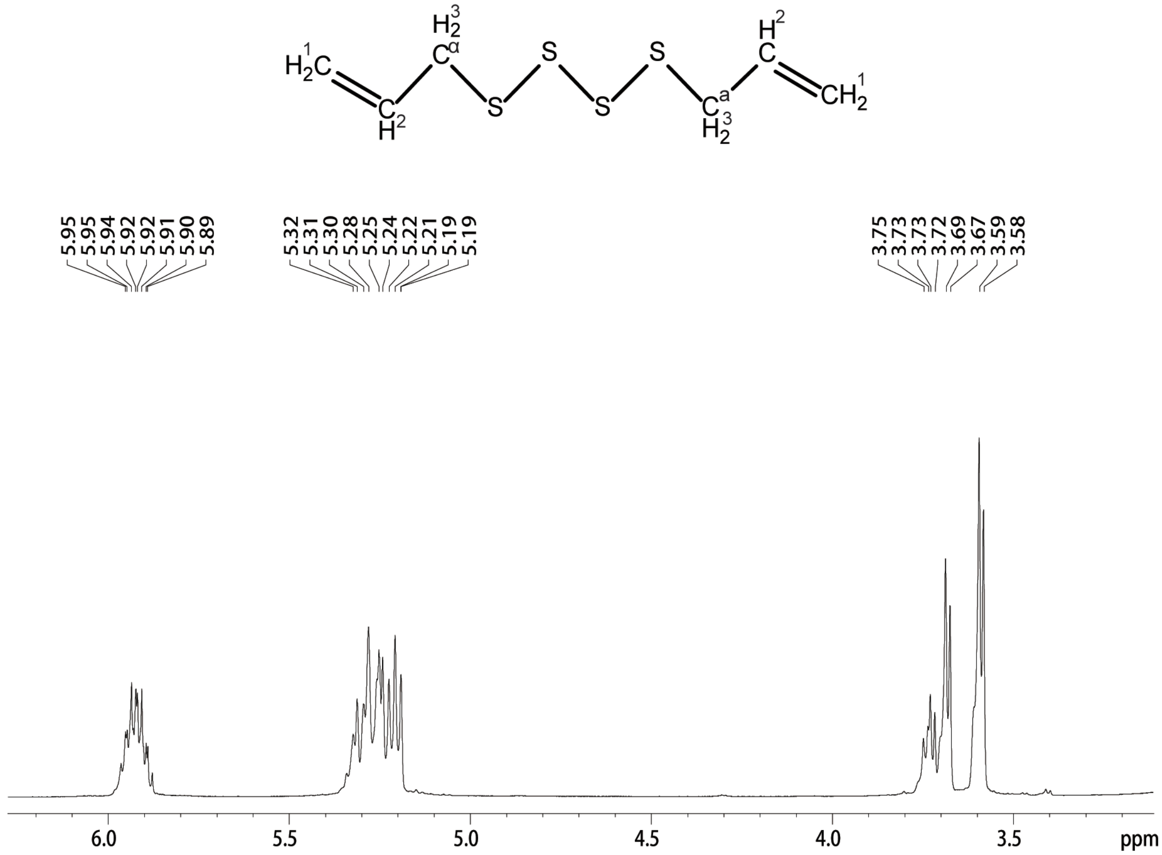

Fig. S6. **^{1}H-NMR spectra of Pey-SSSS-Pey. δ 5.95-5.89 (m, 2H, H^2), δ 5.32-5.19 (m, 4H, H^1), δ 3.75-3.67 (m, 2H, H^3), δ 3.59-3.58 (d, 2H, H^3).**

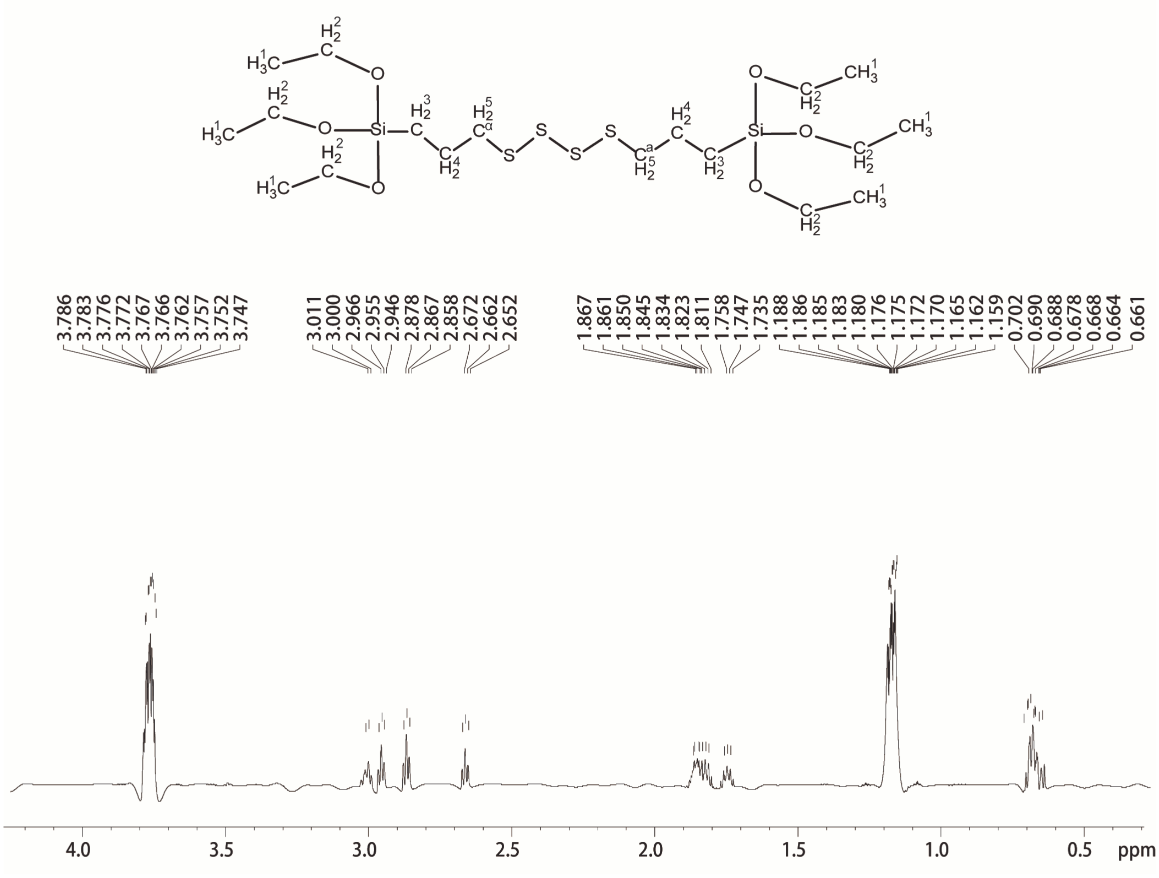

Fig. S7. ^{1}H-NMR spectra of Tsp-SSSS-Tsp. δ 3.78-3.74 (m, 12H, H^2), δ 3.01-2.94; δ 2.87- 2.85; δ 2.67-2.65; (m, 4H, H^5), δ 1.86-1.73 (m, 4H, H^4), δ 1.18-1.15 (m, 18H, H^1), δ 0.70-0.66 (m, 4H, H^3).

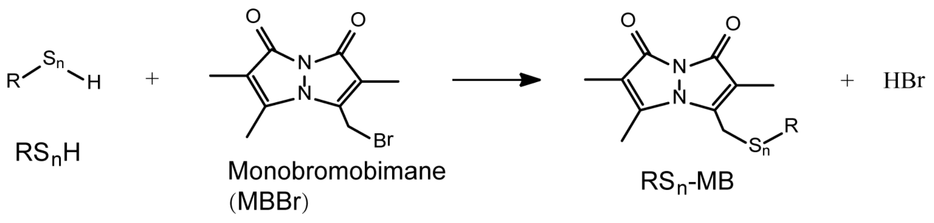

Analyte	Calculated M	Calculated M-H(*M/Z*)	Product ion M-H(*M/Z*)
GSS-MB	529.1296	528.1217	528.1235
GSSS-MB	561.1016	560.0938	560.0949
GSSSG	644.1235	643.1157	643.1181
GSSSSG	676.0956	675.0877	675.0891

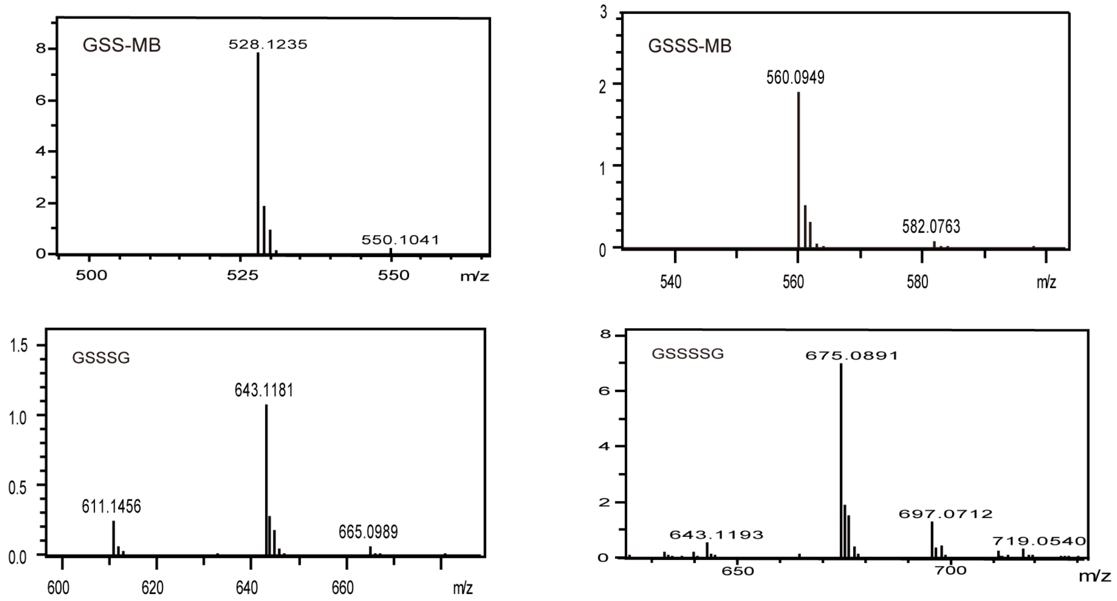

Fig. S8. LC-ESI-MS profiles of MBBr derivatized GSSH and its disproportionation products. The derivatives were separated by HPLC and detected by MS in the negative ion mode.

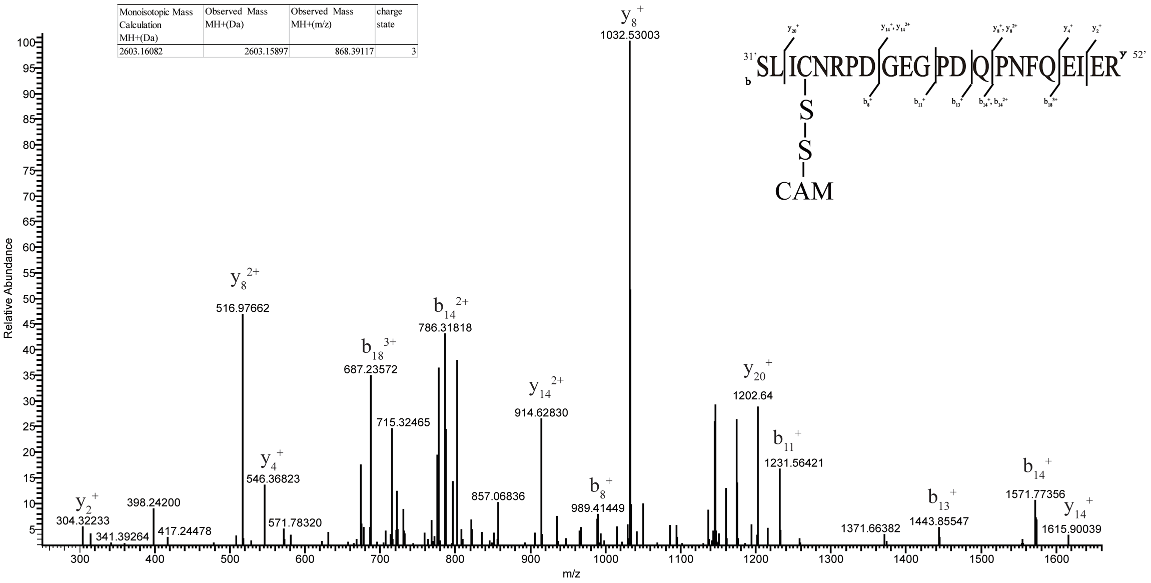

Monoisotopic Mass Calculation MH+(Da)	Observed Mass MH+(Da)	Observed Mass MH+(m/z)	charge state
2603.16082	2603.15897	868.39117	3

Fig. S9. LTQ-Orbitrap tandem mass spectrometry of the Cys34 modification in GSSH-reacted DUF442.

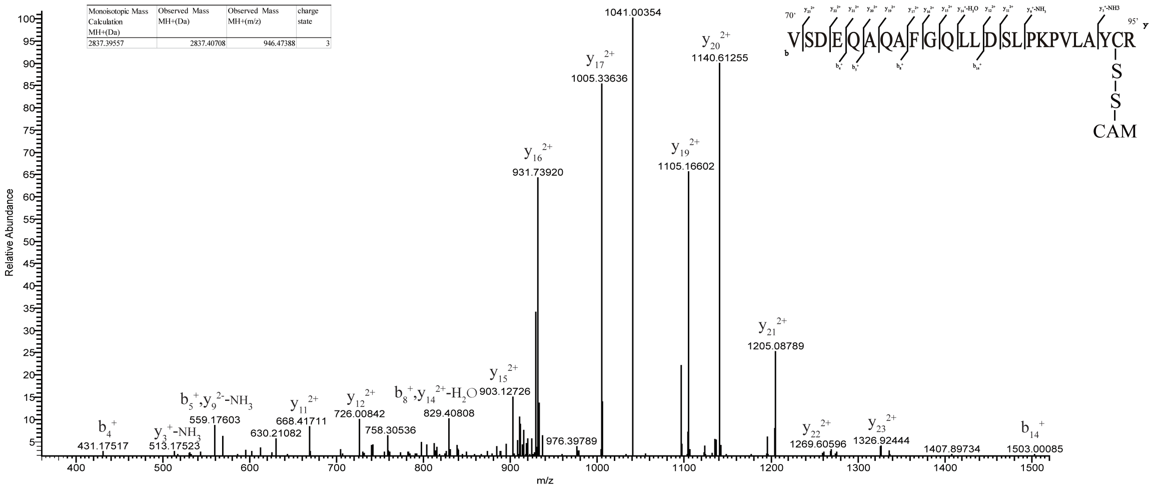

Fig. S10. LTQ-Orbitrap tandem mass spectrometry of the Cys94 modification in GSSH-reacted DUF442.
